# Aging modulates the effects of ischemic injury upon mesenchymal cells within the renal interstitium and microvasculature

**DOI:** 10.1002/sctm.20-0392

**Published:** 2021-05-05

**Authors:** Isaac W. Shaw, Eoin D. O'Sullivan, Angela O. Pisco, Gary Borthwick, Kevin M. Gallagher, Bruno Péault, Jeremy Hughes, David A. Ferenbach

**Affiliations:** ^1^ Centre for Inflammation Research Queen's Medical Research Institute, University of Edinburgh Edinburgh UK; ^2^ Centre for Regenerative Medicine University of Edinburgh Edinburgh UK; ^3^ Department of Renal Medicine Royal Infirmary of Edinburgh Edinburgh UK; ^4^ Chan Zuckerberg Biohub San Francisco California USA; ^5^ Orthopaedic Hospital Research Center and Broad Stem Cell Research Center David Geffen School of Medicine, University of California Los Angeles California USA

**Keywords:** aging, fibrosis, ischemia, kidney, mesenchyme, pericyte

## Abstract

The renal mesenchyme contains heterogeneous cells, including interstitial fibroblasts and pericytes, with key roles in wound healing. Although healing is impaired in aged kidneys, the effect of age and injury on the mesenchyme remains poorly understood. We characterized renal mesenchymal cell heterogeneity in young vs old animals and after ischemia‐reperfusion‐injury (IRI) using multiplex immunolabeling and single cell transcriptomics. Expression patterns of perivascular cell markers (α‐SMA, CD146, NG2, PDGFR‐α, and PDGFR‐β) correlated with their interstitial location. PDGFR‐α and PDGFR‐β co‐expression labeled renal myofibroblasts more efficiently than the current standard marker α‐SMA, and CD146 was a superior murine renal pericyte marker. Three renal mesenchymal subtypes; pericytes, fibroblasts, and myofibroblasts, were recapitulated with data from two independently performed single cell transcriptomic analyzes of murine kidneys, the first dataset an aging cohort and the second dataset injured kidneys following IRI. Mesenchymal cells segregated into subtypes with distinct patterns of expression with aging and following injury. Baseline uninjured old kidneys resembled post‐ischemic young kidneys, with this phenotype further exaggerated following IRI. These studies demonstrate that age modulates renal perivascular/interstitial cell marker expression and transcriptome at baseline and in response to injury and provide tools for the histological and transcriptomic analysis of renal mesenchymal cells, paving the way for more accurate classification of renal mesenchymal cell heterogeneity and identification of age‐specific pathways and targets.

## Graphical abstract

Kidney mesenchyme contains progenitor cells important in disease aetiology yet is poorly characterized with age and lacks research tools. Healthy and ischemia reperfusion injured (IRI) kidneys from old and young mice, analyzed using immunohistochemistry (IHC) and single cell RNA sequencing (scRNAseq), uncovered differences in aged injury response and provided more detailed characterization of kidney mesenchyme. BV = blood vessel.

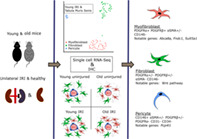

**Significance statement**

The mesenchymal cell compartment plays a key role in kidney disease, but the varied cell types within are poorly defined and the effect of aging on mesenchymal cells is incompletely understood. Here, for the first time the authors perform histological analysis of common mesenchymal markers with accompanying transcriptomic profiling on young and old mice following unilateral ischemia reperfusion injury. This results in a more refined understanding of mesenchymal marker expression as they align with cell subtypes within the mesenchymal compartment. Age associated changes in mesenchymal populations are identified, furthering our understanding of the differences in injury response that occur with age.

## 1 INTRODUCTION

Acute kidney injury (AKI) occurs in approximately 1 in 5 hospital admissions,^1^ and can leave patients with varying degrees of renal fibrosis—an important contributor to the transition to chronic kidney disease (CKD). In addition, the likelihood of a fibrotic outcome post AKI and of progression to CKD increases with age.^3^, ^4^, ^5^, ^6^, ^7^ It is therefore crucial to understand the mechanism of fibrosis following renal injury and the age‐associated factors that drive this.

Bilateral ischemia reperfusion injury (bIRI) is a common model of AKI,^8^ but the long‐term effects on the aged kidney cannot be easily assessed in this model as aged mice tolerate such severe injury poorly leading to a high mortality.^2^, ^9^ In contrast, unilateral ischemia reperfusion injury (uIRI)^8^ transiently interrupts blood flow to one kidney but leaves the contralateral kidney unaffected, thus enabling the study of more unilaterally severe injury than the models of bIRI or uIRI with contralateral nephrectomy.^10^ Importantly, this facilitates the observation of long‐term fibrotic end‐points, increasing the relevance to patients.^11^

Although AKI results in widespread tubular cell injury, the effect upon the interstitium is key for overall kidney outcome: rarefaction of peritubular capillaries with subsequent tissue hypoxia exacerbates the loss of nephrons,^12^ while a progressive fibrotic phenotype in the interstitium is the hallmark of CKD.^13^ These processes are influenced by mesenchymal perivascular/interstitial cells which support the vasculature^14^ and contribute substantially to myofibroblast generation and expansion.^15^ However there has been little research into how age affects perivascular/interstitial progenitor cells,^16^, ^17^ and crucially no research to date into the effect of age on the perivascular/interstitial cell response to injury.

There are two broad types of mesenchymal cells present in the interstitium. “Pericytes” enwrap the microvascular endothelium and are embedded in the capillary basement membrane.^18^, ^19^ “Interstitial fibroblasts” are embedded in and structurally maintain the collagenous extracellular matrix (ECM) of the interstitium.^20^, ^21^, ^22^, ^23^ These fibroblasts are necessarily in close proximity to the capillaries, but are less intimately associated than pericytes. There are likely many subpopulations of pericytes and interstitial fibroblasts, such as the “perivascular fibroblasts” that reside in the collagenous matrix around larger vessels.^24^ In the text below the term “interstitial cell” refers to all mesenchymal cells in the interstitial compartment.

A diverse range of perivascular cell markers, with heterogeneous expression patterns, have been utilized to study renal interstitial cells, such as neural glial 2 (NG2), platelet derived growth factor receptor (PDGFR) ‐β, α‐smooth muscle actin (α‐SMA), and PDGFR‐α. Their heterogeneity in expression is not well characterized, and is indicative of an underlying functional heterogeneity.^17^ Kidney pericyte functional heterogeneity has been demonstrated previously: Gli1^+^ pericytes/perivascular fibroblasts give rise to the majority of myofibroblasts following kidney injury,^25^ and there is a pericyte subset that produce renin.^26^ CD146 is reportedly a ubiquitous human pericyte marker,^27^, ^28^, ^29^ but is poorly characterized in murine kidney. Thus, more detailed characterization of the interstitium is required given the central role of mesenchymal cells in renal injury and recovery.

This work characterizes the interstitial distribution of common perivascular cell markers in the young and aged murine kidney and tests the effects of injury and age on mesenchymal cell phenotypes. This is achieved by multiplexed immunolabeling, which reveals the relative spatial distribution of multiple surface markers within the complex renal architecture, in combination with single cell transcriptomic technology, which provides high‐dimensional information on gene expression within individual cells and allows unbiased clustering into transcriptionally distinct subpopulations. In this work these two technologies independently identify similar subpopulations within the renal interstitium and provide insight into their functional properties through their anatomical localization, their reaction to age and ischemia, and through their gene expression profile.

## 2 MATERIALS AND METHODS

### 2.1 Animals and surgery

Interstitial cell quantification in the cortex and inner stripe was performed on male FVB mice from the National Institute of Aging colony (Charles River, Boston, USA) that were either young (3‐5 months) or old (18 months). IRI surgery for immunolabeling experiments was performed on a separate cohort of FVB mice in the University of Edinburgh Central Bioresearch Services that were either young (3‐5 months) or aged in‐house (18‐24 months). Ischemia time was 25 minutes. For post‐IRI single cell transcriptomics, C57/Bl6 mice were purchased from Jackson Laboratories and bred in‐house before use. Mice were used at 6‐8 weeks of age. Ischemia time was 20 minutes. Mice were culled 28 days post‐surgery. For uIRI surgery animals were anesthetized by inhalation of 2‐4% isofluorane (Merial) and surgery was performed as previously described.^3^ More details in Supplementary Methods.

All animal procedures were approved in advance by the local Animal Welfare Ethical Review Body and performed in accordance with the Animals (Scientific Procedures) Act 1986 (amended in 2012).

### 2.2 Human tissues

Human kidney tissue was collected with prior written informed consent. Ethical approval for the use of human tissues in research was obtained from the South East Scotland Research Ethics Committee.

Tissue was obtained from uninephrectomy operations that were performed following the detection of renal carcinoma. Plugs were taken at maximum distance from neoplasms from the cortex and outer medulla regions. These were fixed in formalin and paraffin embedded, before processing for immunofluorescent staining as described.

### 2.3 Histopathology

Histopathology was performed as previously described.^3^ Kidney halves were fixed overnight in methacarn before paraffin embedding. Five micrometer sections were stained using hematoxylin and eosin, or picrosirius red. For acute tubular necrosis (ATN) scoring at day one, 4‐8 random fields in the outer stripe at 20× magnification were acquired from H&E stained sections. Images were blinded, randomized, and the proportion of tubules with evidence of tubular death was quantified. Fibrosis quantification was performed on eight random fields in the outer stripe of picrosirius red stained sections. % red‐positive area was quantified using ImageJ software.

### 2.4 Immunofluorescence staining

All immunofluorescence staining was performed on methacarn‐fixed paraffin embedded slides as previously described.^3^ The choice of endothelial cell marker (CD31 or CD34) in each case depended on technical considerations such as species of antibody and brightness of labeling. Full details of protocols in Supplementary Methods.

### 2.5 Cell quantification

Positive cell nuclei were putatively identified automatically via co‐localization of antigen signal and DAPI using ImageJ software, followed by manual verification. Only nuclei in the interstitium or on the outer surface of capillaries were deemed positive. Areas of outer stripe were digitally extracted from scans of stained sections. These were either analyzed in their entirety or had 6‐8 high power fields digitally extracted from random locations. Total area quantified for CD146 and PDGFR‐β dual‐labeling was 0.89 mm^2^ per mouse, for α‐SMA and NG2 was 0.17 mm^2^, and for PDGFR‐α and ‐β was 0.09 mm^2^; cell numbers were normalized by total area analyzed. Quantification in the cortex, and outer and inner stripe analyzed 0.35 mm^2^ per region per mouse; cell numbers were normalized by total cross‐sectional area of vasculature analyzed, as determined by CD34 labeling.

### 2.6 Single cell transcriptomics

For animals in the IRI dataset surgery was performed on three animals as described in the Animals & Surgery section. They were culled via exsanguination and cervical dislocation and kidney was dissected out and immediately stored in TPMT on ice before digestion and live/dead FACS sort using DAPI (BioLegend, catalogue 422 801) stain. Single cell libraries from murine kidneys were prepared using a high‐throughput droplet‐based library preparation workflow, Single cell suspensions were prepared as outlined by the 10× Genomics Single Cell 3′ v2 Reagent kit user guide (10x). The sample was then split and sequenced across 4 lanes on a single Illumina flow cell on a NextSeq 550 High Output Kit v2 (Illumina) for 150 cycles at 400 M PE reads comprising of 2x75bp and 8 bp index reads. Alignment was performed by splicing aware aligner STAR 2.5.1b before downstream analysis in the R environment. The Tabula Muris Senis data follows a similar droplet based protocol using the 10× platform with digests from mice of multiple ages and is described in detail in the original manuscript.^30^

Full details of single cell transcriptomic analysis and statistical methods can be found in Supplementary Methods.

### 2.7 Statistical analysis

Data are presented as mean ± SD, or in the case of ratios geometric mean ± 95% CI. For single comparisons in Figure S7B,D,E a two‐tailed two sample *t* test was performed. For ratios (Figures 3H,I,K,L and S9B,C) data were log transformed and difference from 0 was tested by one sample *t* test. Data in Figures 2C‐F; 3G,J; 4D‐F; 5E‐G; S9A; and S12B were analyzed using two‐way ANOVA and Bonferroni corrected post‐tests. Correlations in Figure S10 were analyzed by linear regression. All statistics were performed using Graphpad Prism 5 software. For statistics regarding single cell transcriptomics, see the dedicated section within Supplementary Methods.

## 3 RESULTS

### 3.1 CD146 and PDGFR‐β staining identifies pericytes and interstitial fibroblasts in the murine kidney

**FIGURE 1:**
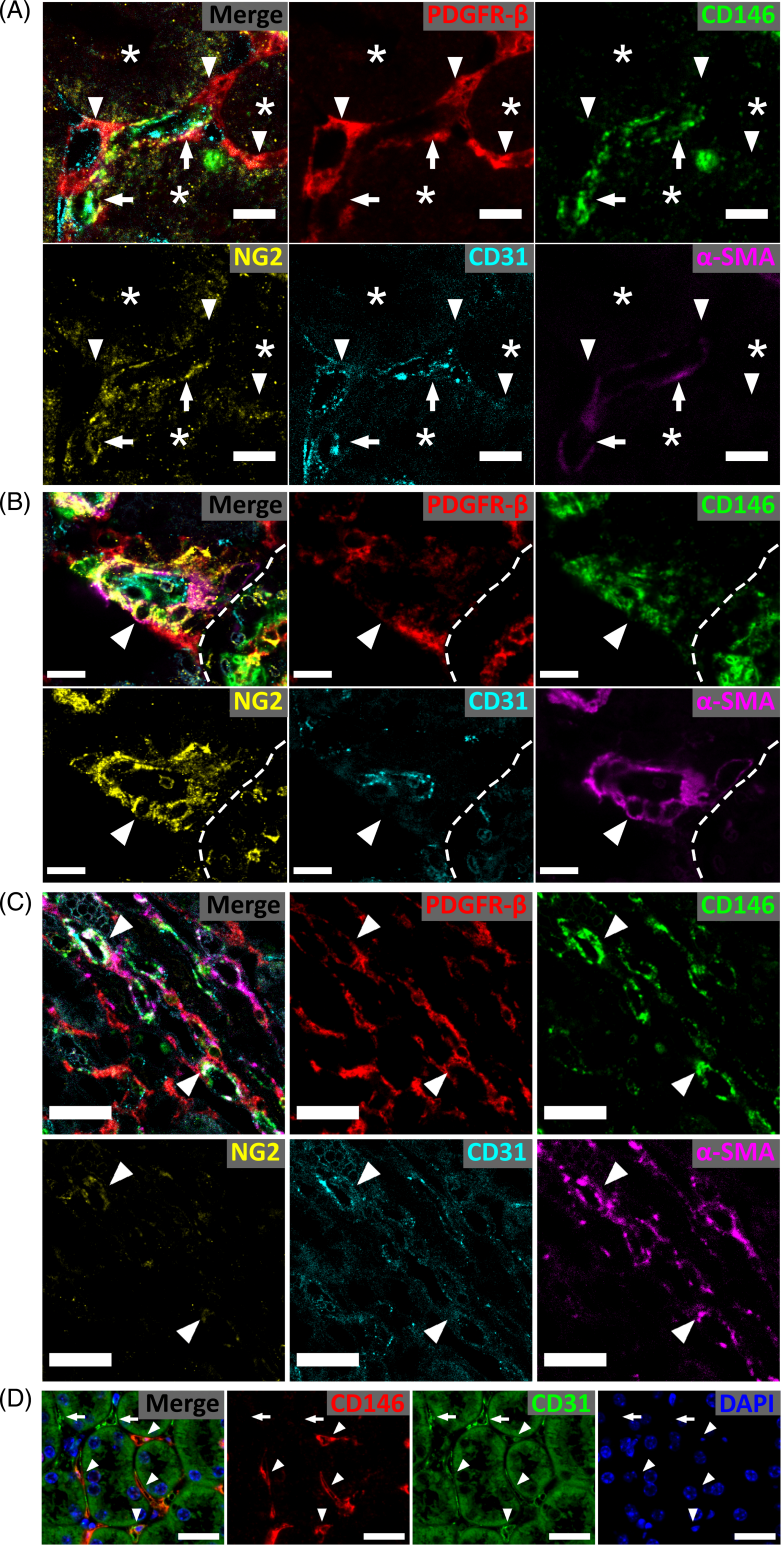
Heterogeneity in the expression and localization of perivascular markers in the kidney. A‐C, Confocal images of healthy kidney labeled for α‐SMA, CD31, CD146, NG2, and PDGFR‐β. A) Peritubular capillary in the cortex. Pericytes positive for multiple markers are visible on the basal aspect of CD31^+^ capillaries (arrows). Note the close association of CD146 with the endothelium. PDGFR‐β^+^ interstitial cells that are negative for other pericyte markers are widely distributed (arrowheads). Similar expression patterns are observed in the outer medulla. Asterisks indicate a tubule. B, Afferent arteriole in the cortex. Smooth muscle‐like pericytes around the arteriole label robustly for CD146, NG2 and α‐SMA, but have low‐to‐absent expression of PDGFR‐β (arrowhead). PDGFR‐β^+^ and PDGFR‐β^+^NG2^+^ cells are present basally to the CD146^+^ layer. CD146 can co‐localize with CD31 on the endothelium. Dashed line marks the border of a glomerulus. C, Image of *vasa recta* from the inner stripe. Arrowheads indicate α‐SMA^+^ perivascular cells around vessels in the *vasa recta*. D, Widefield image of kidney labeled for CD146 and CD31. CD146 is always closely associated with CD31^+^ endothelium (arrowheads). CD31^+^ vessels lacking CD146 coverage are also present (arrows). Scale bars (A‐C) 10 μm; (D) 50 μm

**FIGURE 2:**
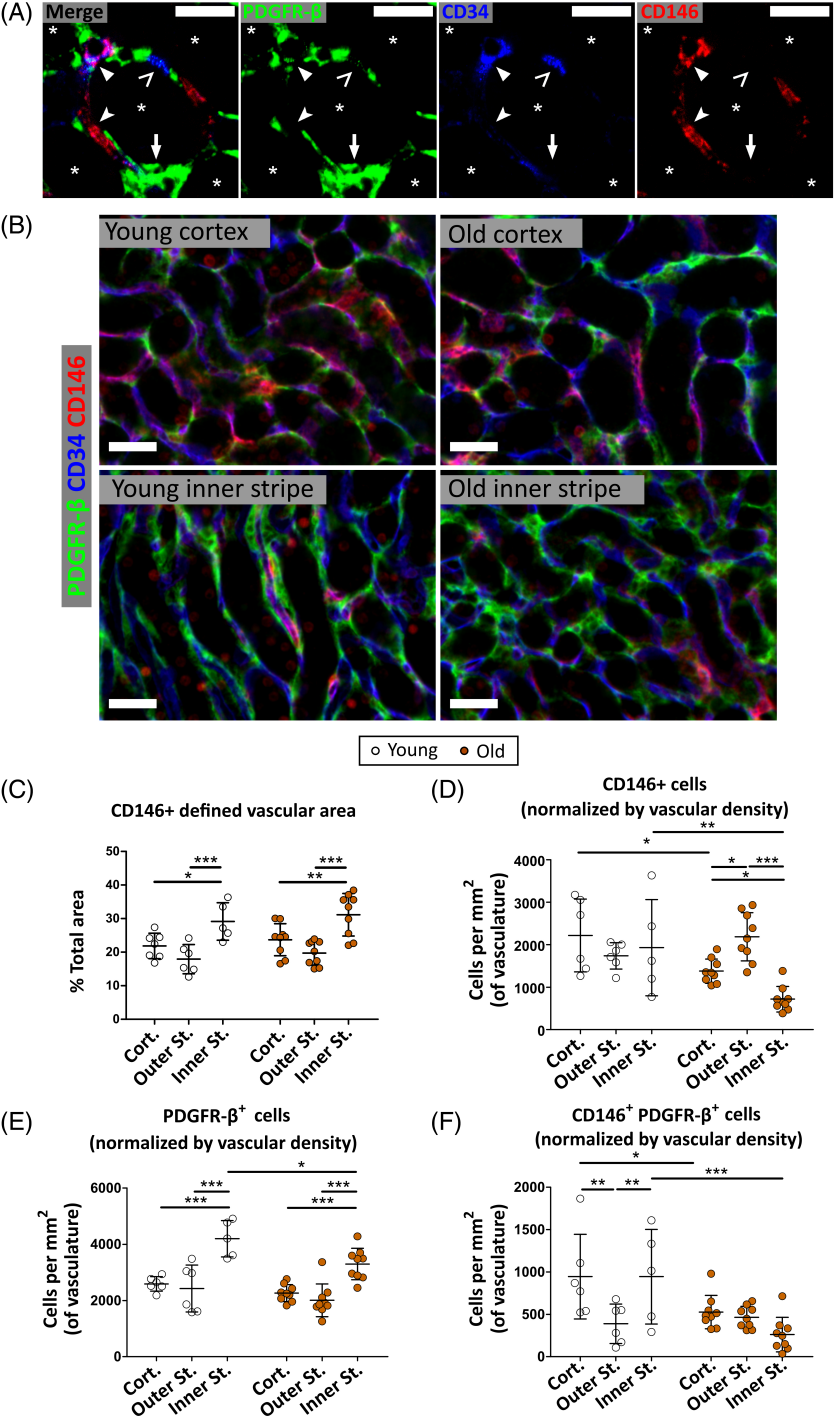
Age‐ and region‐linked differences in interstitial cell subpopulations as defined by CD146 and PDGFR‐β expression. A, Confocal microscopy image of CD146, PDGFR‐β, and CD34 labeling in the mouse kidney, outer stripe region, with a 1.5 μm optical section. Cells single positive for PDGFR‐β (arrow), CD146 (notched arrowhead) and CD34 (open arrowhead) are visible, along with endothelial co‐localizations of CD146 and CD34 (filled arrowhead) and CD146 and PDGFR‐β (not shown). Tubules labeled with an asterisk. B, Widefield images of CD146, PDGFR‐β and CD34 labeling of young and old uninjured cortex and inner stripe (DAPI also present but not shown). Scale bars (A) 15 μm; (B) 25 μm. C, Quantification of CD34^+^ vascular area. D‐F, Quantification of CD146^+^PDGFR‐β^−^ (D), CD146^−^PDGFR‐β^+^ (E), and CD146^+^PDGFR‐β^+^ (F) interstitial nuclei in the cortex, outer stripe and inner stripe of young and old mice, normalized per mm^2^ of CD34^+^ vasculature. *P* values from two‐way ANOVA are shown for each graph. *P < .05; **P < .01; ***P < .001 from Bonferroni post hoc tests. N = 5‐8 per group

Multiple markers are used to label perivascular/interstitial cells in murine renal studies (Table 1). To test their usefulness together as perivascular/interstitial cell markers in murine kidney, sections were labeled for α‐SMA, PDGFR‐β, NG2, CD146, and the endothelial cell marker CD31 (Figures 1 and S1‐S3). Separate sections were also labeled for PDGFR‐β, CD146, and endothelial cell marker CD34 (Figure 2). CD146^+^CD31^−^ and CD146^+^CD34^−^ perivascular populations were identified around peritubular capillaries of the cortex and outer medulla (Figures 1A,C; 2A; S1; and S2). CD146^+^ perivascular cells were often co‐labeled with NG2 and PDGFR‐β (Figures 1A,B; S1 and S3). In contrast, no cells with convincing pericyte morphology were labeled CD146^−^NG2^+^ or CD146^−^PDGFR‐β^+^, indicating that CD146 identifies all murine renal pericytes. CD146^+^ endothelial cells were also observed (Figures 1B, 2A, and S3) however many peritubular capillaries lacked CD146 expression (Figure 1D). In human kidney biopsies, CD146 labeled kidney pericytes, and a subset of endothelial cells (Figure S4A‐D).

**TABLE 1:** Properties of perivascular cell surface markers and their previous use in murine renal studies

Interstitial surface marker	Other names	Description	Use in murine renal studies
PDGFR‐α	CD140a	Receptor for PDGF‐A, ‐B, and ‐C.^31^ Linked to a fibrotic interstitial cell phenotype in studies of muscle.^32^, ^33^	Studies of glomerular and interstitial fibrosis^34^
PDGFR‐β	CD140b	Receptor for PDGF‐A, ‐B, and ‐D.^31^ Commonly used pericyte marker in multiple tissues including kidney. Involved in pericyte recruitment during angiogenesis/vasculogenesis. Brain studies suggest that PDGFR‐β dependent binding to CD146 in pericyte progenitors facilitates their coverage of endothelial cells.^35^	Common pericyte marker. Studies of glomerular and interstitial fibrosis^34^
CD146	Melanoma‐associated cell adhesion molecule (MCAM)	Receptor for laminin‐α‐4 (endothelial basement membrane),^36^ and interacts with actin cytoskeleton^37^ and calcium signaling.^38^ Can dimerize with VEGFR2^39^ and PDGFR‐β (see above) for angiogenesis roles. Historically used as an endothelial maker; ubiquitous marker of pericytes in human tissue.^27^	Rarely used^25^
NG2	Chondroitin sulfate proteoglycan 4	Proposed roles in detecting extracellular matrix components and relaying signals to the cytoskeleton.^40^ Necessary for full pericyte coverage of retinal vessels.^41^ In human it is specifically not expressed on venular pericytes.^27^ Expression lost during pericyte quiescence and regained upon stimulation (eg, following injury).^24^	Common pericyte marker
α‐SMA	α‐actin‐2	Role in cell contraction. Presence on stromal cells indicates a collagen producing myofibroblast.^42^ Also present on contractile pericytes, for example, on arterioles and descending *vasa recta*.^17^	Extensively used as myofibroblast marker

A substantial population of PGDFR‐β^+^CD146^−^NG2^−^α‐SMA^−^ cells were present. They were distributed in the interstitial ECM and did not show the same intimate association with vessels as CD146^+^PDGFR‐β^−^ and CD146^+^PDGFR‐β^+^ pericytes (Figures 1A‐C and 2A, and S1‐S3) indicating that PDGFR‐β^+^ cells lacking CD146 expression are interstitial fibroblasts. NG2 expression was occasionally observed on these interstitial fibroblasts (PDGFR‐β^+^CD146^−^NG2^+^α‐SMA^−^) localized to the periphery of larger vessels, basally to the CD146^+^α‐SMA^+^ pericyte/vascular smooth muscle cell (vSMC) layer (Figures 1B and S3). α‐SMA localized to SMC‐like pericytes on larger vessels such as afferent arterioles (Figures 1B and S3), and descending *vasa recta* (Figures 1C and S2) where it overlapped substantially with CD146 and NG2, but was undetectable on capillary pericytes and interstitial fibroblasts (Figures 1A and S1). This contrasted with human, where α‐SMA was present on the majority of capillary pericytes (Figure S4A‐D).

Based on these observations, labeling for CD146 and PDGFR‐β alongside an endothelial cell marker, such as CD31, effectively identifies pericytes and distinguishes them from interstitial fibroblasts in the baseline murine kidney. In endothelial marker negative cells, CD146^+^ (±PDGFR‐β^+^) staining identifies pericytes, while a CD146^−^PDGFR‐β^+^ phenotype identifies interstitial fibroblasts (summarized in Figure S5).

### 3.2 Reductions in pericyte and fibroblast numbers in the renal interstitium occur with age

Reductions in vascular area and pericyte numbers with age have been reported.^16^ To determine the effects of age on perivascular/interstitial cell numbers, kidney sections from young (3‐5 months) and old (18 months) mice were triple‐labeled for PDGFR‐β, CD146, and the endothelial cell marker CD34^43^, ^44^ (Figure 2A,B) and the relative abundances of CD146^+^PDGFR‐β^−^(CD34^−^), CD146^−^PDGFR‐β^+^(CD34^−^), and CD146^+^PDGFR‐β^+^(CD34^−^) interstitial cells were quantified in the cortex, outer stripe, and inner stripe regions (Figure 2C‐F). CD146^+^PDGFR‐β^+^ pericytes were a minority subset of each population, namely 27‐33% of the CD146^+^ population and 7‐28% of the PDGFR‐β^+^ positive population (Figure S6). This indicates that the major portion of cells in the total PDGFR‐β^+^ interstitial population are not pericytes but rather interstitial fibroblasts. This analysis revealed a general increase in CD146^−^PDGFR‐β^+^ interstitial cells (ie, interstitial fibroblasts) from cortex to inner stripe (Figure 2E). The number of CD146^−^PDGFR‐β^+^ interstitial fibroblasts in the inner stripe decreased from young to aged kidneys (Figure 2E). There was also a decrease in CD146^+^PDGFR‐β^+^ and CD146^+^PDGFR‐β^−^ cells (ie, pericytes) in the cortex and inner stripe, but not the outer stripe (Figure 2D,F). These results indicate that there is a loss of pericyte coverage on renal vessels with age in the cortex and inner stripe, along with a loss of interstitial fibroblasts in the inner stripe.

### 3.3 Initial injury is not significantly worse in aged vs young mice following severe unilateral ischemia/reperfusion

Following 25 minutes of warm uIRI young and old mice were culled at one‐ and 28 days post‐ischemia. Subsequent investigations unless otherwise indicated focused on the outer stripe of the outer medulla, as this is the region most susceptible to tubular cell injury following ischemia and relatively little injury was observed in the cortex and inner stripe. ATN scoring at day one post‐IRI indicated that a significant injury was inflicted (Figure S7A,B). However, there was no difference in ATN score between the ischemic kidneys of old and young mice (Figure S7B), indicating that the degree of initial tubular injury was equivalent between ages.

### 3.4 Old animals have increased interstitial fibrosis compared with young both before and after unilateral ischemia reperfusion injury

To test whether old animals have a greater fibrotic response following a similar initial ischemic injury, young and old animals were culled at 28 days post‐IRI and fibrosis was quantified in the outer stripe by picrosirius red staining (Figure S7C‐E). There was significantly more fibrosis in the old kidneys of the contralateral and ischemic groups (Figure S7D,E). There was no obvious change in fibrosis in contralateral kidneys between days one and 28 (Figure S8).

### 3.5 Dynamic changes in CD146 ^+^ PDGFR‐β^+^ pericyte numbers following injury that are absent in aged animals

**FIGURE 3:**
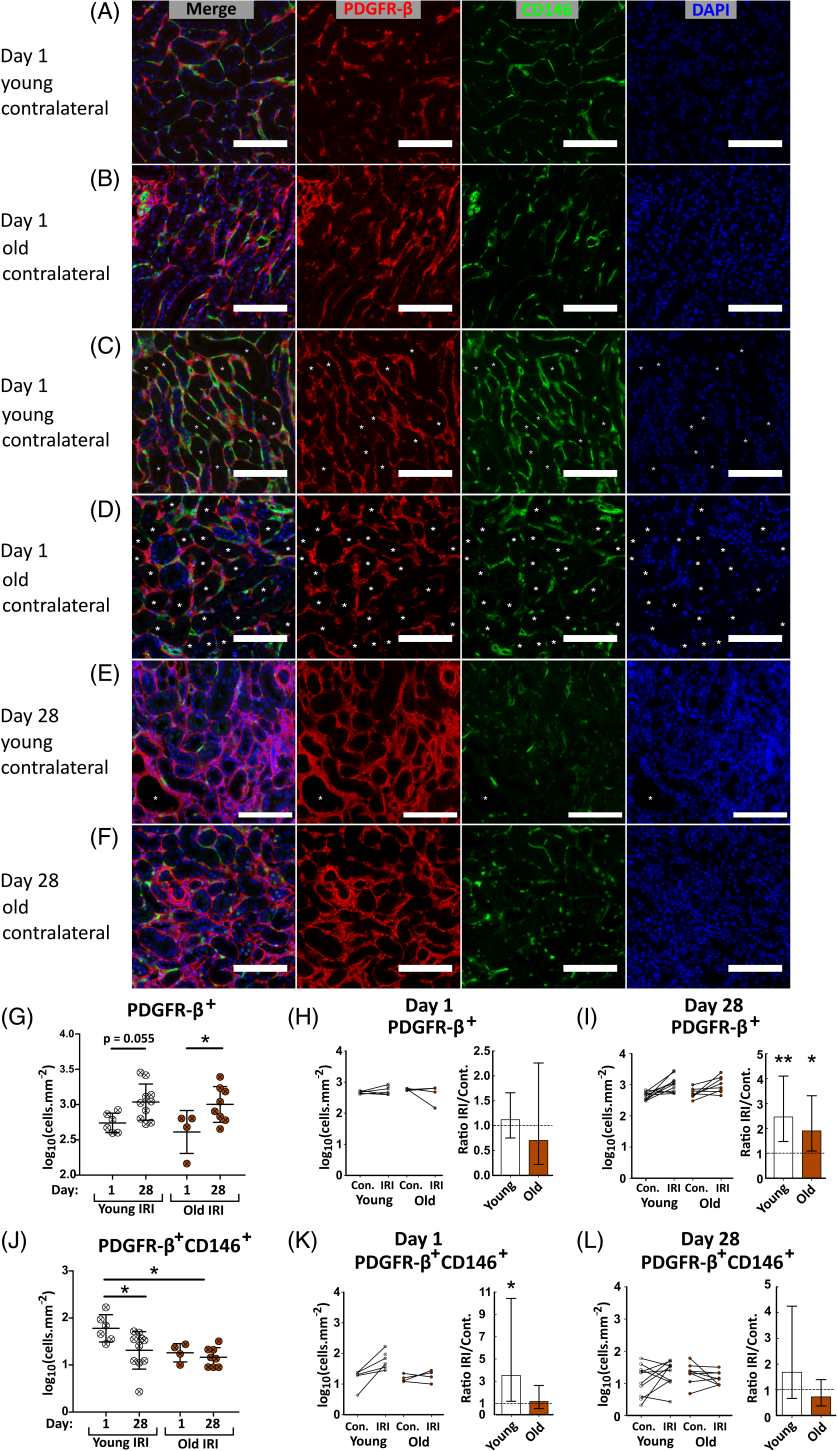
Quantification of CD146^+^ and PDGFR‐β^+^ interstitial cells in the outer stripe at days one and 28 following unilateral ischemia reperfusion injury. A‐F, Outer stripe region of kidneys from the IRI injury experiment labeled for CD146 (green), PDGFR‐β (red), and with DAPI (blue). Contralateral kidneys at day one (A,B) and ischemic kidneys at days one (C,D) and 28 (E,F) are shown, as indicated. Asterisks indicate denuded tubules. Scale bar = 100 μm. G and J, Quantification of PDGFR‐β^+^CD146^−^ (G), or PDGFR‐β^+^CD146^+^ (J), interstitial cells in the outer stripe of ischemic kidneys across injury time course. Means were compared by two‐way ANOVA, *P* values displayed. *P < .05; **P < .01 in Bonferroni post hoc tests. Lines indicate mean ± SD. I‐L, Ratio comparisons between contralateral and ischemic kidneys at day 1 (H,K) and day 28 (I,L) post‐injury. Log ratios significantly different from one by one sample *t* test indicated with asterisks: *P < .05; **P < .01. Bars show geometric mean ± 95% CI. N = 4‐10 per group. Con., contralateral kidney; IRI, ischemic kidney

To observe the mesenchymal cell response following uIRI, CD146^+^PDGFR‐β^−^, CD146^−^PDGFR‐β^+^ and CD146^+^PDGFR‐β^+^ interstitial cells were quantified at one‐ and 28 days post‐IRI in young and old contralateral and ischemic kidneys (Figures 3 and S9). CD146^−^PDGFR‐β^+^ interstitial fibroblasts were significantly increased in ischemic kidneys of young and old animals at day 28 compared with contralateral kidneys and day‐one ischemic kidneys (Figure 3G‐I). No difference was detected between ages (Figure 3G).

In young ischemic kidneys strong CD146 labeling was observed around denuded tubules (identified morphologically and by lack of nuclei) at day 1 (Figure 3C). In old animals CD146 was not so obviously activated and localized (Figure 3D). CD146^+^ pericytes may be separated into subtypes with or without PDGFR‐β expression. CD146^+^PDGFR‐β^−^ pericyte numbers were unchanged in ischemic compared with contralateral kidneys in both age groups (Figure S9B,C), however young animals exhibited a marked increase in CD146^+^PDGFR‐β^+^ pericytes at day one in ischemic kidneys in contrast to old (Figure 3J,K). CD146^+^PDGFR‐β^+^ pericyte numbers in ischemic kidneys were equivalent to contralateral levels at both ages by day 28 (Figure 3L). These data demonstrate a transient increase in a CD146^+^PDGFR‐β^+^ pericyte subtype early in the wound healing response of young animals that is absent in old animals.

### 3.6 NG2 and PDGFR‐α identify interstitial cell populations associated with fibrosis and more abundant in old kidney

**FIGURE 4:**
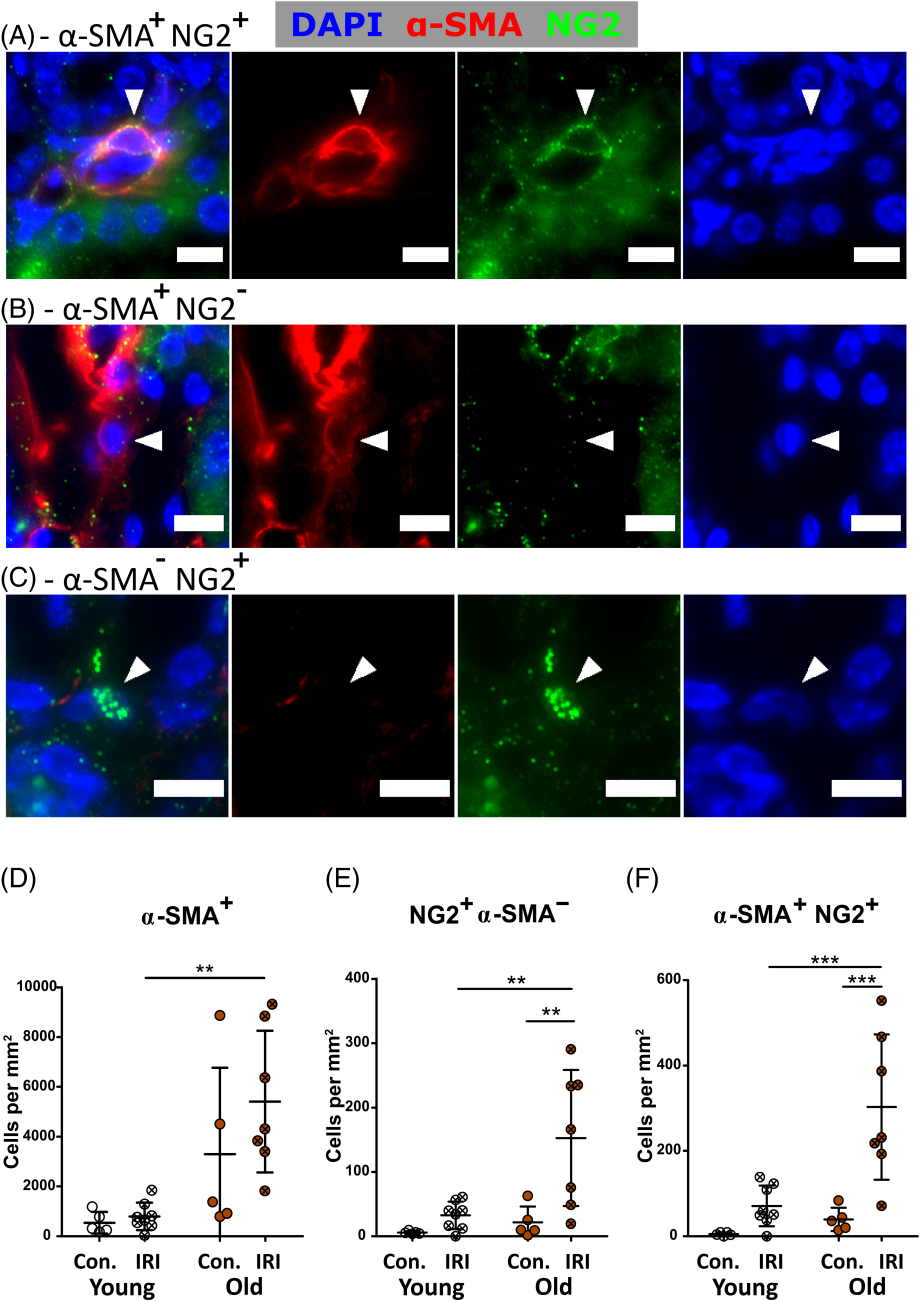
NG2^+^ and α‐SMA^+^ cell quantification in the outer stripe of contralateral kidneys, and ischemic day‐28 kidneys following unilateral ischemia reperfusion injury. A‐C, NG2 and α‐SMA labeling in the outer stripe of contralateral kidney and ischemic day‐28 post‐injury kidney. Examples of NG2^+^α‐SMA^+^ (A), NG2^−^α‐SMA^+^ (B), and NG2^+^α‐SMA^−^ (C) cells are shown (arrowheads). Scale bars = 10 μm. D‐F, Quantification of NG2^+^α‐SMA^+^ (D), NG2^+^α‐SMA^−^ (E), and α‐SMA^+^ (F) nuclei observed. Comparisons made using two‐way ANOVA. *P < .05; **P < .01; ***P < .001 by Bonferroni post hoc tests. N = 5‐7 per group. Con., contralateral kidney; IRI, ischemic kidney

Renal fibrosis is mediated by myofibroblasts, commonly defined as α‐SMA^+^ interstitial cells,^24^, ^25^, ^43^ and the NG2 pericyte marker has been associated with pericytes/interstitial cells activated by pathological processes.^24^, ^45^ To quantify myofibroblasts and investigate their relationship with NG2^+^ cells, α‐SMA^+^, α‐SMA^+^NG2^+^, and α‐SMA^−^NG2^+^ interstitial cells were quantified at day 28 post‐ischemia (Figure 4).

At 28 days post‐IRI, NG2^+^α‐SMA^+/−^ cells were significantly more abundant in old ischemic kidneys than at baseline and were significantly higher in number than in corresponding young kidneys (Figure 4D,E), suggesting a more active pathological phenotype in old kidneys following ischemic injury. Supporting this idea, there was an increase in NG2 expression in the inner medulla of old kidneys at day 28 post‐IRI that was absent in young kidneys (Figure S12), indicating that activation of NG2 expression was more widespread in old kidneys.

Old ischemic kidneys had significantly more α‐SMA^+^ myofibroblasts than young (Figure 4F) suggesting a more active fibrotic phenotype. Furthermore, in day‐28 post‐IRI ischemic kidneys NG2^+^α‐SMA^+/−^ cell numbers correlated more closely with fibrosis area than α‐SMA^+^ myofibroblast numbers (Figure S10D‐F). Previous work showed that not all α‐SMA^+^ cells express collagen,^24^ and α‐SMA expression on other renal cell types such as macrophages.^46^ We thus asked whether more specific myofibroblast markers exist.

**FIGURE 5:**
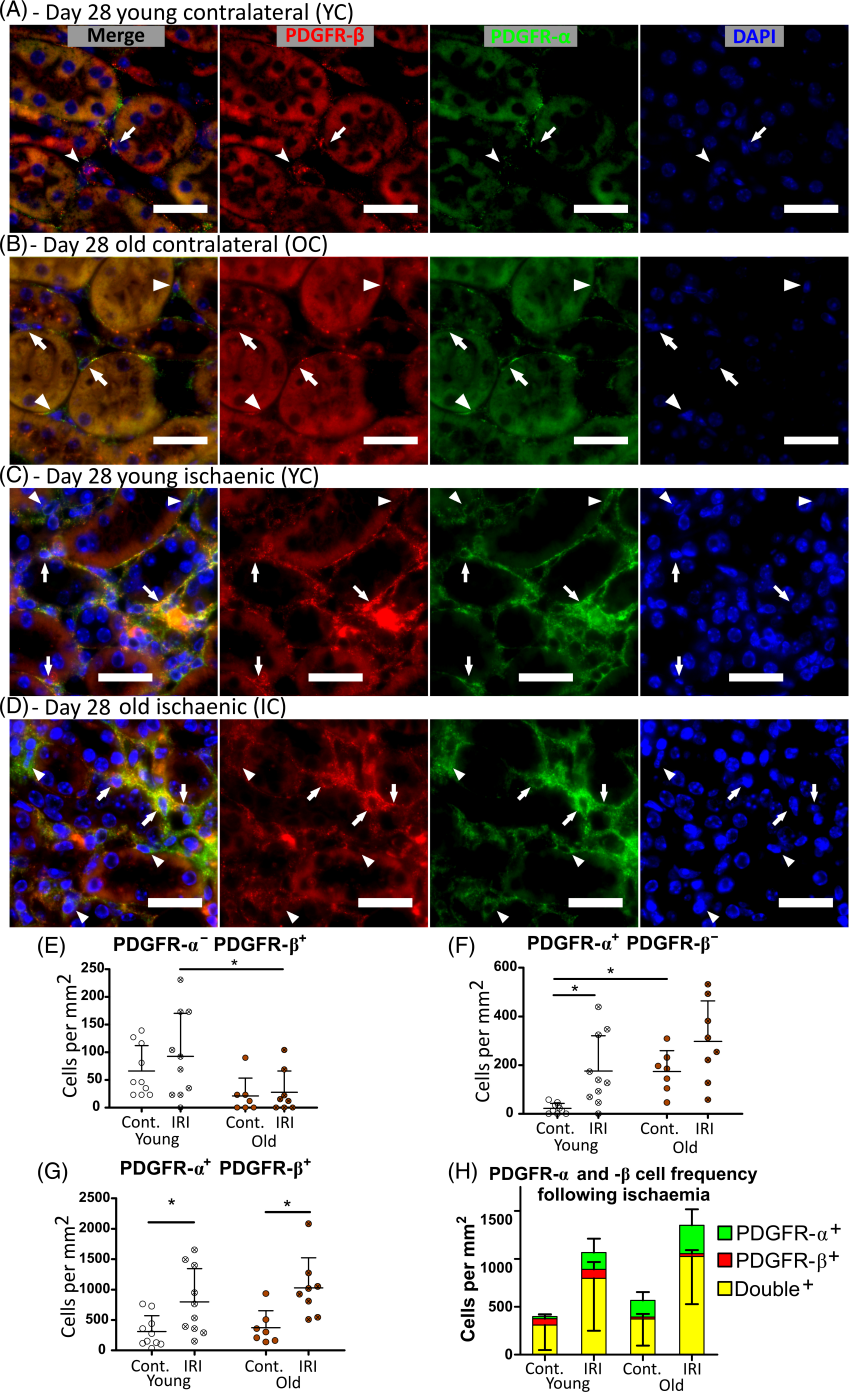
PDGFR‐α^+^ and ‐β^+^ cell quantification in the outer stripe at day 28 following unilateral ischemia reperfusion injury. A‐D, PDGFR‐α (green) and PDGFR‐β (red) labeling in the outer stripe of young (A,C) and old (B,D) contralateral (A,B) and ischemic (C,D) kidneys at day 28 post‐injury. PDGFR‐α^+^‐β^+^ (arrows), PDGFR‐α^+^‐β^−^ (arrowheads), and PDGFR‐α^−^‐β^+^ (notched arrowheads) nuclei are observed. Scale bars = 25 μm. E‐G, Quantification of the numbers of PDGFR‐α^−^‐β^+^ (E), PDGFR‐α^+^‐β^−^ (F), and PDGFR‐α^+^‐β^+^ (G) nuclei observed. Comparisons analyzed by two‐way ANOVA, *P < .05 in Bonferroni post hoc tests. (H) PDGFR‐α^+^ and PDGFR‐β^+^ Comparison of the relative numbers of each interstitial subtype in the different experimental groups. N = 7‐10 per group. Cont., contralateral kidney; IRI, ischemic kidney

PDGFR‐α is a mesenchymal marker associated with a fibrotic phenotype in skeletal and cardiac muscle.^32^, ^33^ PDGFR‐α shows little co‐localization with CD146, NG2, or α‐SMA in healthy kidney (Figure S11A‐D) but substantial overlap with PDGFR‐β (Figure 5). PDGFR‐α^+^‐β^−^, PDGFR‐α^−^‐β^+^, and PDGFR‐α^+^‐β^+^ interstitial cell numbers in the outer stripe of day 28 kidney tissue were quantified (Figure 5). PDGFR‐α was observed mainly on interstitial fibroblasts, whereas PDGFR‐α^−^‐β^+^ cells only occurred rarely and in perivascular locations (Figure 5A,B). This suggests that PDGFR‐α^+^‐β^+/−^ cells are interstitial fibroblasts, whereas PDGFR‐β^+^ cells that lack PDGFR‐α expression are pericytes. PDGFR‐α^+^‐β^+^ cells increased significantly with ischemia at both ages and correlated more strongly with fibrosis than α‐SMA^+^ cells (Figures 5G,H; S10D,I), suggesting they may together label fibrosis‐producing myofibroblasts. There was substantial, but not absolute, co‐localization of PDGFR‐α and α‐SMA in injured kidneys (Figure S11E). In contralateral kidneys the PDGFR‐α^−^‐β^+^ pericyte population tended to be lower in old than young animals and was significantly lower following ischemia, consistent with a loss of pericytes with age (Figure 5E,H). In contralateral kidneys the PDGFR‐α^+^‐β^−^ population was significantly higher in old than young (Figure 5F,H). Interestingly, young day‐28 ischemic kidneys had also acquired a population of PDGFR‐α^+^‐β^−^ cells, suggesting PDGFR‐α^+^‐β^−^ cells are a subtype of interstitial fibroblasts arising in response to pathological stimuli (Figure 5F,H). This suggests a persistent activated state of interstitial cells 28 days post‐IRI and in aged kidney at baseline.

### 3.7 Single cell transcriptomics independently identifies pericytes, fibroblasts, and myofibroblasts in young and old kidney

**FIGURE 6:**
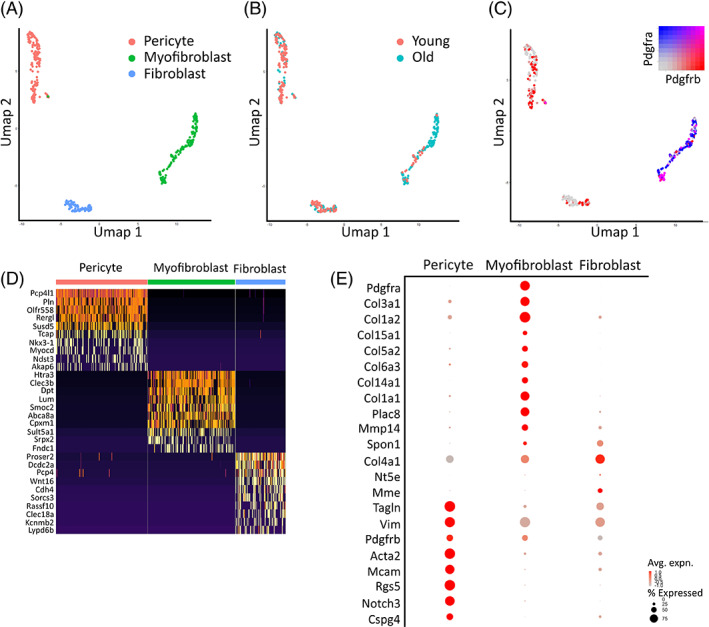
Single cell RNA sequencing identifies three distinct mesenchymal‐like populations resembling pericytes, fibroblasts, and myofibroblasts. A‐C, Umap reduction of 400 murine mesenchymal cells colored by (A) shared nearest neighbor (SNN) allocated cluster; (B) age of mouse as young (1‐3 months, pink) or old (18, 21, and 30 months, turquoise); or (C) blended log_10_ expression level of genes *Pdgfra* (blue) and *Pdgfrb* (red). D, Heatmap demonstrating the top 10 differentially expressed genes by fold change per cluster, calculated using Wilcoxon signed‐rank test. The color scheme is based on z‐score distribution. E, Expression of selected genes across clusters. Dot size represents the percentage of cells in each cluster expressing the gene; dot color represents average log_10_ gene expression

To investigate the transcriptional identity and functional phenotype of cells identified through imaging studies we performed a new analysis on the renal compartment of the Tabula Muris Senis (TMS) kidney single cell RNA sequencing (scRNAseq) dataset (Figures S13 and S14).^30^ This dataset is comprised of male and female C57BL/6JN mice across six age groups, ranging from 1 month (the equivalent of human early childhood) to 30 months (the equivalent of a human centenarian). Given the variable expression of common mesenchymal markers demonstrated above and elsewhere,^17^ we deemed that a whole digest dataset, as opposed to for example FACS sorting, would give a more unbiased selection of all mesenchymal cells present in the kidney. From 21 647 starting cells (from all age groups) we isolated 400 cells with mesenchymal identity as detailed in the methods. This mesenchymal cell yield is similar to other whole kidney digest scRNAseq experiments.^47^, ^48^ These were enriched in mesenchymal‐identity genes (*Tagln*, *Acta2*, *Col1a1/2*, *Adamts2*, *Mfap5*) (Figures S13C‐E and S14). These cells formed three discrete clusters, with differential gene expression that corroborated imaging studies (Figure 6A,D, Supplementary Data 1) allowing for classification. The myofibroblast population expressed multiple collagens, and co‐expressed *Pdgfra* (PDGFR‐α) and *Pdgfrb* (PDGFR‐β) (Figure 6C,E). We classified the age of a mouse as “young” (aged 1‐3 months) or as “old” (18, 21, and 30 months). Cells from old animals appeared to be overrepresented in the myofibroblast population (Table 2, Figure 6B) consistent with our immunofluorescence dataset. Pericytes/vSMC had high expression of *Mcam* (CD146), *Acta2* (α‐SMA), and *Cspg4* (NG2), but lacked *Pdgfra* (Figure 6E). The third population appeared to be quiescent fibroblasts. They expressed genes typical of a functional renal fibroblast such as *Slc6a6*, *Tsc22d1*, and *Mid1ip1*, but had relatively lower collagen expression (Supplementary Data 1, Figure 6E). Differentially expressed genes also showed enrichment in components of the Wnt pathway such as *Ahi1*, *Dcdc2a*, *Fzd3*, *Fzd4*, *Gsk3b*, *Lrp6*, *Lypd6b*, *Wnt5a*, and *Wnt16* (Supplementary Data 1, Figure 6D, Table S2).

**TABLE 2:** Proportion of cells in each cluster of the tabula muris senis dataset, and all clusters combined, that is derived from each age group

Age group	All clusters combined	Pericyte cluster	Myofibroblast cluster	Fibroblast cluster
All young (1–3 months)	48.8%	71.9%	17.7%	60.9%
1 month	28.0%	43.8%	9.2%	32.2%
3 months	20.8%	28.1%	8.5%	28.7%
All old (18‐30 months)	51.3%	28.1%	82.3%	39.1%
18 months	14.8%	10.6%	22.2%	9.2%
21 months	9.3%	11.9%	7.2%	8.0%
30 months	27.3%	5.6%	52.9%	21.8%

### 3.8 Independent single cell transcriptomics of post‐ischemic kidney identifies pericytes, fibroblasts, and myofibroblasts in young mice

**FIGURE 7:**
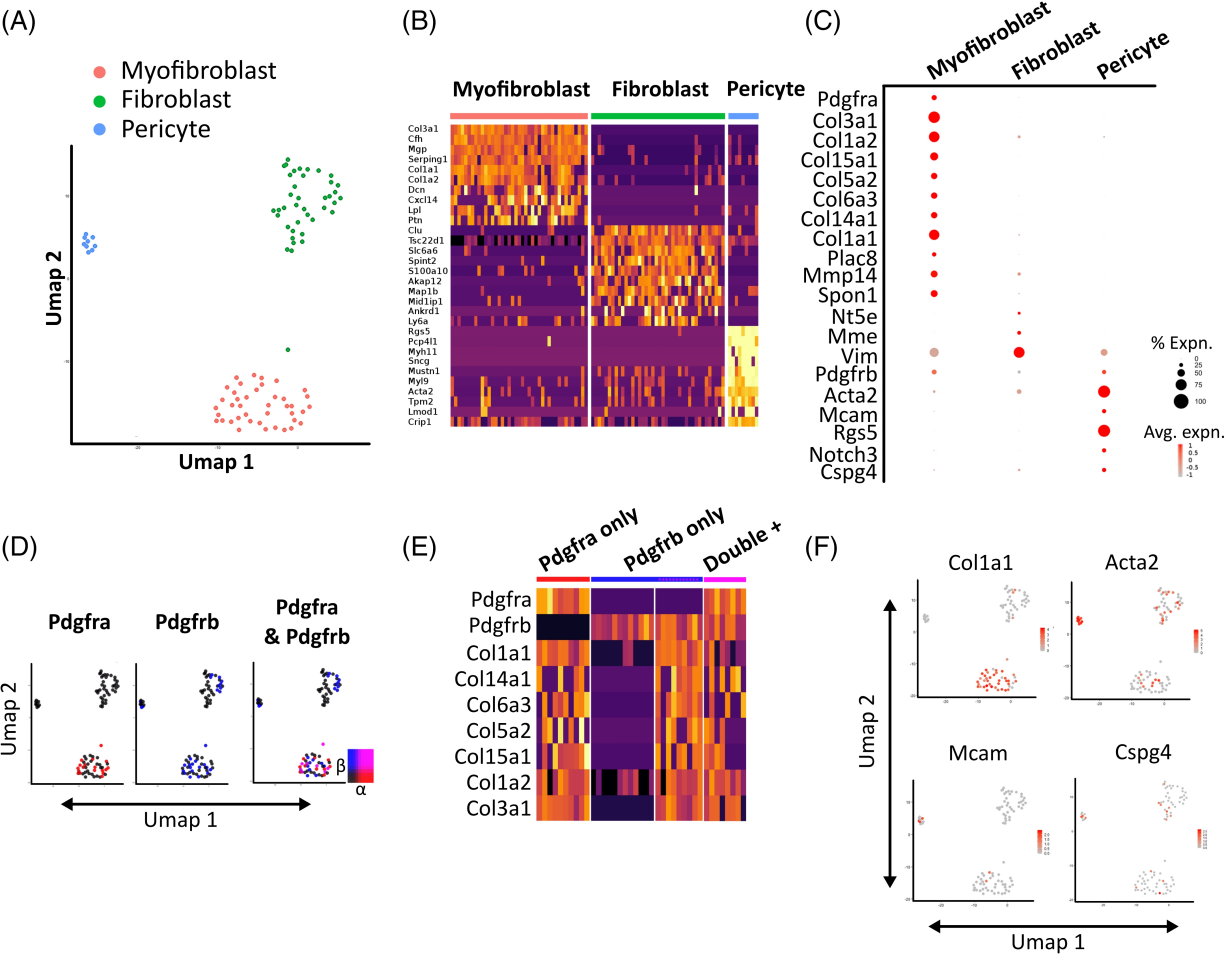
Single cell RNA sequencing of kidney 28 days post‐IRI identifies three distinct mesenchymal‐like populations resembling pericytes, fibroblasts, and myofibroblasts. A, Umap reduction of 90 murine mesenchymal cells 28 days post‐IRI colored by shared nearest neighbor (SNN) allocated cluster. B, Heatmap demonstrating the top 10 differentially expressed genes by fold change per cluster, calculated using Wilcoxon signed‐rank test. The color scheme is based on z‐score distribution. C, Expression of selected genes across clusters. Dot size represents the percentage of cells in each cluster expressing the gene, color represents average log_10_ gene expression. D, Featureplot showing co‐expression of *Pdgfra*/*Pdgfrb* projected onto Umap cell coordinates. Color scale represents relative scale of gene expression, blend threshold set to 0.25. E, Heatmap showing key fibrotic genes grouped by presence of *Pdgfra*/*Pdgfrb* transcripts. Asterisks denote cells suspected of being *Pdgfra* positive due to transcriptional similarities to the dual positive cluster. The color scheme is based on z‐score distribution. F, Gene expression of selected marker genes projected onto Umap plot

To observe the effects of IRI on mesenchymal transcriptomic profiles, we performed unbiased scRNAseq on digests of whole injured kidneys 4 weeks post‐IRI (Figure S15). From 2931 starting cells we isolated 90 cells with high confidence mesenchymal identity which again formed three distinct clusters, consistent with pericyte, myofibroblast and fibroblast populations and sharing differential gene expression similar to the TMS dataset (Figure 7A‐C, Supplementary Data 2). Using an automated “anchor gene” approach (Supplementary Methods), 89/90 cells in the IRI data were assigned a classification derived from the TMS transcriptomes which corresponded exactly to the classification we had manually assigned “de novo” (Figure S16A,B). Additionally, we used the SCMAP approach to project cell classifications from the TMS scRNAseq data set onto individual cells from the IRI data. The mapping is shown in the Sankey plot, demonstrating high concordance between the automated and manual classifications (Figure S16C). The high congruence between the IRI dataset and the TMS dataset gave us confidence in the validity of our identified cell populations, despite our comparatively lower mesenchymal cell numbers. The %‐yield of total cells was also similar between experiments (1.8% and 3.1% for TMS and IRI datasets, respectively). As with aging, post IRI we found *Pdgfra* and *Pdgfrb* co‐localized only in the high collagen‐expressing myofibroblast population, whereas *Acta2* was most highly expressed in the pericyte/vSMC population (Figure 7D‐F).

## 4 DISCUSSION

The renal mesenchyme has vast importance in disease and consists of a complex assortment of cells with disparate roles and biologies.^17^ Despite this there is a lack of understanding, and awareness, of renal mesenchymal heterogeneity that inevitably has a detrimental impact on the design and interpretation of studies. It is therefore imperative to thoroughly characterize this cell compartment and provide tools for its effective study. Here we have identified mesenchymal subtypes both transcriptomically and histologically, developed methods to distinguish these subtypes histologically, and determined subtype localization at baseline and with injury and aging.

Using scRNAseq, we have identified three major subtypes of renal mesenchymal cells, namely pericytes/vSMCs, fibroblasts, and myofibroblasts. The same populations were identified in two independently performed sequencing experiments which encompass physiological aging (TMS dataset)^30^ and the effect of injury (IRI dataset). Importantly these studies were performed without pre‐selection based on a transgene or surface marker, allowing us to avoid selection bias due to incomplete marker coverage (a problem highlighted by our histology studies) or variations in transgene expression. Our analysis represents an improvement on previous unbiased approaches, where only a generic “fibroblast” population was identified in mouse whole‐kidney scRNAseq, and “pericyte/vSMC” and “interstitial” populations were identified in human single nucleus RNA sequencing.^47^, ^48^ Our ability to identify myofibroblast populations was likely due to our inclusion of aged and injured kidney tissue.

Many previous studies in kidney have used PDGFR‐β as a marker of “pericytes.”^14^, ^15^, ^17^, ^24^, ^43^, ^49^, ^50^, ^51^, ^52^ The data presented here demonstrate that PDGFR‐β, used alone,^14^, ^49^, ^50^, ^51^, ^52^ is an inappropriate pericyte marker in murine kidney because the majority of its labeling identifies interstitial fibroblasts. In our experience CD146 is the most specific marker for distinguishing pericytes from other renal mesenchymal cells and is also highly sensitive, as is the case in other human and mouse organs.^27^, ^35^, ^53^ We propose a combination of CD146, PDGFR‐β and endothelial cell labeling as the most accurate method of identifying and distinguishing pericytes and interstitial fibroblasts in histological sections of murine kidney. Under this model, within the non‐endothelial population pericytes are CD146^+^ (±PDGFR‐β labeling), and interstitial fibroblasts are CD146^−^PDGFR‐β^+^ (see Figure S5). In the future, prospective pericyte markers identified through our scRNAseq analysis should be tested, such as Purkinje cell protein 4‐like protein 1 (*Pcp4l1*) which was in the top differentially expressed genes for pericytes in both the IRI and TMS datasets.

We have also identified a combination of PDGFR‐α and PDGFR‐β labeling as a potentially superior method of identifying myofibroblasts. This is through corroboration of histological data, in which this population increased substantially with ischemia (Figure 5G) and correlated strongly with fibrosis extent (Figure S10I), and scRNAseq data in which only the collagen‐expressing myofibroblast population expressed both the *Pdgfra* and *Pdgfrb* transcripts (Figures 6C,E and 7C‐E). In line with this PDGFR‐α is a marker of fibrotic populations and progenitors in other tissues such as muscle, skin, and multiple visceral organs.^32^, ^54^, ^55^ It is often in association with PDGFR‐β^25^, ^32^, ^56^ which is itself reported a myofibroblast progenitor marker.^57^ Furthermore, a contemporaneous study recently published also identified PDGFR‐α^+^PDGFR‐β^+^ cells as the major ECM producers in human and mouse renal fibrosis.^58^ These two proteins are therefore emerging as reliable fibrotic population markers across multiple organ systems. Other prospective myofibroblast markers may be identified from scRNAseq data. Of the top differentially expressed genes for myofibroblasts three, *Abca8a*, *Fndc1*, and *Sult5a1*, have probable intracellular expression (www.uniprot.org) and thus represent markers to investigate in future.

Aging is one of the largest risk factors for renal disease,^7^ yet the effect of age on the mesenchymal cell response to injury has not to our knowledge been investigated beyond 12 months in mice.^59^ Following IRI, the response of old kidney mesenchyme differed to young in several ways. First, increased fibrosis with age was detected in line with previous ischemia studies.^2^, ^3^, ^59^ Second, transient increases in pericyte numbers and CD146 labeling intensity seen in young animals at day 1 post‐IRI were not observed in old. Third, at day 28 there was substantially more ischemia‐linked subtypes in old animals, especially NG2^+^ populations. Indeed, analysis of mesenchymal subtypes suggests that the aged mesenchyme at baseline is in a “post‐ischemic” state. For example, NG2^+^α‐SMA^−^, NG2^+^α‐SMA^+^, and PDGFR‐α^+^‐β^−^ cells increased with ischemia in both age groups, and while they were difficult to detect in young contralateral kidneys, in old contralateral kidneys they were reasonably abundant. Taken together, this points to a situation in which the old kidney has a subdued acute response to injury, chiefly in the pericyte population, but an extended or non‐resolving long‐term reaction within fibroblast and myofibroblast populations. Non‐resolving fibrosis is well documented with aging,^60^, ^61^ and previous studies have shown an impaired proliferative response in kidney in the acute phase of injury, although none have targeted pericytes specifically.^2^, ^9^, ^62^, ^63^ It is possible that age related senescence is a factor in both these observations^2^, ^64^; however, further testing is required to ascertain whether and how these differences impact the progression of renal disease.

Closer analysis of scRNAseq gene expression data leads to some interesting findings. First, expression of *Acta2* (α‐SMA), the classical myofibroblast marker, was more substantial in the pericyte/vSMC than in myofibroblast population, as observed elsewhere.^65^, ^66^, ^67^, ^68^, ^69^, ^70^ It has been shown that only 75% of α‐SMA^+^ cells express collagen,^24^ and removal of significant numbers of α‐SMA^+^ cells by inhibiting TGF‐β signaling had only a small effect on fibrosis.^46^ Given these findings, there is reason to suppose that *Acta2*/α‐SMA is a poor marker of collagen‐producing myofibroblasts in kidney. Although pericyte clusters expressed *Acta2* transcripts strongly, not all pericytes had detectable histological α‐SMA expression. There are several possible explanations, including the sensitivity limits of immunological detection, the well documented discrepancy between RNA and protein levels, or the pericyte/SMC cluster being dominated by vSMCs and strongly α‐SMA^+^ pericytes such as those in the arterioles and descending *vasa recta*.

Second, our transcriptomic “Fibroblast” populations likely represent a heterogeneous group of cells. They differentially express genes typical of renal interstitial fibroblast, such as *Tsc22d1* (sodium excretion^71^), *Slc6a6* (osmoregulation^72^), and *Mid1ip1* (lipid synthesis^73^, ^74^); but also genes with links to pericyte biology including *Akap12* (endothelial tight junctions^75^), *Ankrd1 (*SMC‐expressed^76^), *Map1b* (formation of processes^77^, ^78^). This could be due to the fact that capillary and venular pericytes have morphological similarities with interstitial fibroblasts and thus may cluster with them, separate from arteriolar pericytes/SMCs.^31^ Notably, fibroblasts expressed many Wnt pathway‐related genes. Wnts play a prominent role in kidney development^79^ and disease,^80^, ^81^ and may be a key route of communication between renal mesenchyme and epithelium during homeostasis and disease.^82^

Our study likely does not capture the full extent of mesenchymal heterogeneity in the kidney. Multiple subtypes of mesenchymal cells have been identified in the past including ladder‐like medullary fibroblasts,^21^ erythropoietin‐expressing fibroblasts,^83^ renin^+^ juxtaglomerular pericytes,^26^ stem‐like or MSC‐like progenitors,^84^, ^85^ Gli1^+^ myofibroblast progenitors,^25^ as well as general mesenchymal subtypes such as adventitial fibroblasts^86^, ^87^ and others.^17^, ^45^ The findings presented here complement these previous investigations into renal mesenchyme subpopulations. Gli1^+^ cells are major myofibroblast progenitors in kidney, with a surface phenotype of CD146^−^NG2^−^PDGFR‐α^+^PDGFR‐β^+^,^25^ which is analogous with our PDGFR‐α^+^PDGFR‐β^+^ myofibroblast population. Lin et al. reported that collagen expression did not fully correlate with α‐SMA expression and also noticed an induction of NG2 with disease.^24^ Finally, renal mesenchymal populations with “stem‐like” or “MSC‐like” properties, such as multilineage differentiation potential in vitro and ability to integrate into the kidney when delivered in an injury setting, have been identified in multiple studies using various combinations of surface marker proteins Sca‐1, c‐Kit, CD24, CD29, CD44, CD73, CD90, and CD105.^84^, ^88^, ^89^, ^90^, ^91^, ^92^, ^93^ We have found that transcription of these surface markers is distributed across all of our scRNAseq populations (not shown). Given that MSCs can be generated in vitro from both pericytes and adventitial fibroblasts, and that these MSC‐like populations are often selected by digests and plating of whole kidneys, it is likely that they have a mixed origin within the kidney.

In future, renal mesenchymal heterogeneity can be probed more deeply by increasing the yield of cells used in transcriptomic analysis. Besides improved cell dissociation protocols, fluorescent fate mapping using pan‐mesenchymal gene drivers such as FoxD1^15^ or myelin protein zero^94^ should label the majority of mesenchyme and provide an efficient method of cell sorting. Alternatively, subpopulations can be interrogated individually using markers such as those used here. This approach has been recently performed with PDGFR‐α and PDGFR‐β, revealing multiple subpopulations within this compartment.^58^ Much work is still required to investigate the effect of age on renal mesenchyme, and whether and how differences such as those identified here impact on the progression and treatment of disease.

## 5 CONCLUSION

Despite the heterogeneity in perivascular marker expression in the renal interstitium, there is correlation between marker expression and anatomical location, and clear injury‐ and age‐linked differences have been identified. Further research is required to elucidate the full extent and functional implications of renal interstitial cell heterogeneity, and the intriguing questions raised in this study of marker heterogeneity should stimulate such investigations.

## CONFLICT OF INTEREST

The authors declared no potential conflicts of interest.

## AUTHOR CONTRIBUTIONS

I.W.S.: conception and design, collection and/or assembly of data, data analysis and interpretation, manuscript writing, final approval of manuscript; E.D.O.: collection and/or assembly of data, data analysis and interpretation, manuscript writing, final approval of manuscript; A.O.P.: provision of study material (data), collection and/or assembly of data, final approval of manuscript; G.B.: performance of animal surgery; K.M.G.: provision of study material; B.P. and J.H.: conception and design, data interpretation, manuscript writing, final approval of manuscript; D.A.F.: conception and design, performance of animal surgery, data interpretation, manuscript writing, final approval of manuscript.

## Supporting information


**Figure S1**. Co‐localizations in Figure 1A. A, 5‐color image from Figure 1A. B, Same image. All possible pairwise combinations of channels are shown. Column titles indicate the channel that is in magenta in that column, and row titles indicate the channel that is in green in that row. When magenta and green display as white when superimposed. Arrows, CD31^+^ capillaries. Arrowheads, PDGFR‐β^+^ interstitial cells. Asterisk, a tubule.
**Figure S2**. Co‐localizations in Figure 1C. A, 5‐color image from Figure 1C. B, Same image. All possible pairwise combinations of channels are shown. Column titles indicate the channel that is in magenta in that column, and row titles indicate the channel that is in green in that row. When magenta and green display as white when superimposed. Arrowheads, *vasa recta* α‐SMA^+^ perivascular cells.
**Figure S3**. Co‐localizations in Figure 1B. A, 5‐color image from Figure 1C. B, Same image. All possible pairwise combinations of channels are shown. Column titles indicate the channel that is in magenta in that column, and row titles indicate the channel that is in green in that row. When superimposed, magenta and green display as white. Arrowhead, arteriolar pericytes. Dashed line, boundary of glomerulus.
**Figure S4**. CD146 and α‐SMA label perivascular cells in human kidney. Human kidney biopsy tissue was labeled for CD146 (green), α‐SMA (red), nuclei (DAPI, grays) and endothelial cells (*Ulex europaeus* agglutinin, blue). A, small caliber vessel. Pericytes are double positive for CD146 and α‐SMA (arrowheads). Endothelial cells have detectable CD146 labeling (arrow). Scale bar = 10 μm. B, As well as CD146^+^α‐SMA^+^ pericytes (notched arrowhead), pericytes single positive for CD146 (arrow) are also observed. α‐SMA single positive cells (arrowhead) are always located basally to the CD146^+^ layer. Bar = 15 μm. C, Non‐fibrotic region. Double positive pericytes (arrows) and occasional CD146^−^α‐SMA^+^ interstitial fibroblasts are observed. Bar = 25 μm. D, Area of fibrosis. CD146^+^α‐SMA^+^ pericytes are closely associated with vessels (arrows). There has been an expansion of interstitial fibroblasts, but these are all CD146^−^α‐SMA^+^. Bar = 25 μm.
**Figure S5**. Schematic detailing how to use CD146, PDGFR‐β and an endothelial marker to identify pericytes and interstitial fibroblasts in murine kidney.
**Figure S6**. Stacked display of interstitial cell quantification data from Figure 2. Fainter colors indicate data from aged animals. This display highlights how the proportion of PDGFR‐β^+^ cells that are CD146^−^ (red) far outweighs the proportion that are CD146^+^ (yellow). If CD146 positivity (in a perivascular location) identifies a pericyte, as proposed, then the majority of PDGFR‐β^+^ cells in the renal interstitium are not pericytes.
**Figure S7**. Histological injury scoring at day one and fibrosis scoring at day 28 following unilateral ischemia‐reperfusion injury. A, Low magnification views of day 1 contralateral and ischemic kidneys of both ages, as indicated, stained with hematoxylin and eosin (H&E). Dashed line outlines the outer stripe. Bar = 500 μm. B, Quantification of acute tubular nephropathy (ATN), defined as the percentage of tubules in the outer stripe exhibiting necrotic epithelial cells, at day one post injury. Ischemic groups compared by Student's *t* test and no difference detected. C, Low magnification views of picrosirius red (PSR) stained kidneys at day 28 following injury. Dashed line outlines the outer stripe. Bar = 500 μm. C and D, Quantification of % red positive area following PSR staining in young and old contralateral (D), and ischemic (E), kidneys. The old kidneys are more scarred in both cases. Compared using unpaired two‐way *t* test, *P < .05; ***P < .001. N = 4‐10 per group.
**Figure S8**. Comparison of fibrosis in contralateral kidneys at days one and 28. Low magnification representative views of PSR stained sections of contralateral kidney from days one (A,C) and 28 (B,D) post‐injury. There is no obvious difference in fibrosis between days in either young (A,B) or old (C,D) groups.
**Figure S9**. Quantification of CD146^+^PDGFR‐β^−^ cells in the outer stripe at one and 28 days post‐ischemia. A, CD146^+^PDGFR‐β^−^ cell numbers in young and old ischemic kidneys at day one and 28 post‐ischemia. Groups compared by two‐way ANOVA, results displayed. **P < .01 in Bonferroni post hoc tests. B and C, The ratio of CD146^+^PDGFR‐β^−^ cells between contralateral and ischemic kidneys is shown at day one (B), and day 28 (C). Bars show mean ± 95% CI. Log ratios were tested for their difference from 0 using a one sample *t* test. Although CD146^+^ cell numbers appear decreased in young at day 28 vs day 1 (A), there is no difference compared with the contralateral kidney at either time point (B,C). Likewise, old day‐28 ischemic kidneys have significantly more CD146^+^ cells compared with the respective young group, but there is no significant difference to the contralateral old kidneys at this timepoint. The differences observed in the ANOVA are thus likely due to the chance interaction of multiple smaller undetectable differences. N = 4‐10 per group.
**Figure S10**. Correlation of perivascular subtype numbers with area of fibrosis in young and old day 28 post‐injury kidneys. Cell frequency is plotted against % fibrosis area. Blue dots and lines correspond to young animals, red dots and lines correspond to old animals, and black lines are for the combined populations. Solid lines indicate a significant relationship. *R* ^2^‐ and *P*‐values are given for significant relationships (*P* < .05), and also for nonsignificant relationships where *P* < .1. A‐C, Values from CD146 and PDGFR‐β dual labeling. D‐F, Values from α‐SMA and NG2 dual labeling. G‐I, Values from PDGFR‐α and ‐β dual labeling. Relationships analyzed by linear regression. This plot contains data from Figures 3, 4, 5.
**Figure S11**. Co‐labeling of PDGFR‐α with NG2, CD146 and α‐SMA in healthy and injured kidney. Kidney from either healthy (A‐D) or 28 days post‐IRI kidney (E) was labeled for PDGFR‐α and either NG2 (A), CD146 (B), or α‐SMA (C‐E). A, Little interstitial co‐localization of NG2 with PDGFR‐α is observed. Single positives for NG2 (arrowheads) and PDGFR‐α (arrows) are indicated. B, Little to no co‐localization of CD146 with PDGFR‐α is observed. Interstitial PDGFR‐α labeling (arrows) and perivascular CD146 labeling (arrowheads) is indicated. C and D, Little co‐localization of PDGFR‐α (D, arrow) with α‐SMA in healthy tissue, either in α‐SMA^+^ arterioles (C, arrow) or interstitial cells (C and D, arrowheads). E, In 28 days post‐IRI tissue, there is extensive co‐localization of α‐SMA and PDGFR‐α. However, single positive cells for PDGFR‐α (arrowhead) and α‐SMA (arrow) are also observed. Scale bars 15 μm.
**Figure S12**. NG2 labeling in the inner medulla. A, NG2 expression in the inner medulla of young and old contralateral kidney, and day 28 ischemic kidneys. Scale bar = 25 μm. B, Quantification of NG2^+^ area in the interstitium at day 28 post‐IRI. Analyzed by two‐way ANOVA, *** < .001 in Bonferroni post hoc tests. N = 4‐10 per group.
**Figure S13**. Context of mesenchymal populations within the Tabula Muris Senis single cell RNA sequencing dataset. A‐D, Umap of 21 647 individual transcriptomes from the droplet‐based kidney analysis within the Tabula Muris Senis dataset^36^ colored by (A) shared nearest neighbor (SNN) allocated cluster; (B) age group; (C) log_10_ *Pdgfra* expression level; (D) log_10_ *Pdgfrb* expression level. E, Expression of selected typical mesenchymal genes across clusters. Dot size represents the percentage of cells in each cluster expressing the gene, color represents average gene expression log_10_.
**Figure S14**. Differentially expressed genes in Tabula Muris Senis dataset clusters. Heatmap of top 10 marker genes by fold change in each cluster calculated using Wilcoxon signed‐rank test. The color scheme is based on z‐score distribution.
**Figure S15**. Characteristics of single cell RNA sequencing of whole kidney 28 days post‐IRI. A, Umap of 2931 single cell transcriptomes colored by shared nearest neighbor (SNN) allocated cluster. B, Heatmap demonstrating the top 10 differentially expressed genes by fold change per cluster, calculated using Wilcoxon signed‐rank test. The color scheme is based on z‐score distribution. Asterix denotes cluster 8, which was taken forward for further sub‐clustering to identify mesenchymal cells.
**Figure S16**. Analysis of cluster equivalence between Tabula Muris Senis dataset and post‐IRI dataset. A, UMAP of 490 single cells from integrated IRI and TMS dataset. Colored by initially assigned classification in the individual datasets, shape shows dataset of origin. B, Heatmap showing classification prediction score for each cell in the IRI dataset based on transcriptional anchors derived from TMS classifications. C, Sankey diagram demonstrating the mapping of cells within each initial cluster of the IRI dataset to predicted classifications based on the TMS dataset, as calculated by SCMAP.
**Table S1**. Properties of perivascular cell surface markers and their previous use in murine renal studies.
**Table S2**. Proportion of cells in each cluster of the tabula muris senis dataset, and all clusters combined, that is derived from each age group.

**Appendix S2**. Supporting Information Data 1.

**Appendix S3**. Supporting Information Data 2.

## Data Availability

scRNAseq data produced and analyzed in this work has been deposited in the Gene Expression Omnibus (GEO), which can be found at https://www.ncbi.nlm.nih.gov/geo/. The accession number is GSE140010. Data from the Tabula Muris Senis dataset has been published.^30^
