# Supplementary material for: Aging modulates the effects of ischemic injury upon mesenchymal cells within the renal interstitium and microvasculature
Source: Stem Cells Transl Med. 2021 May 5;10(8):1232–48. doi: 10.1002/sctm.20-0392 (PMC8284778; doi:10.1002/sctm.20-0392)
Supplement: Supplementary file 1 — Figure S1. Co‐localizations in Figure 1A. A, 5‐color image from Figure 1A. B, Same image. All possible pairwise combinations of channels are shown. Column titles indicate the channel that is in magenta in that column, and row titles indicate the channel that is in green in that row. When magenta and green display as white when superimposed. Arrows, CD31+ capillaries. Arrowheads, PDGFR‐β+ interstitial cells. Asterisk, a tubule. Figure S2. Co‐localizations in Figure 1C. A, 5‐color image from Figure 1C. B, Same image. All possible pairwise combinations of channels are shown. Column titles indicate the channel that is in magenta in that column, and row titles indicate the channel that is in green in that row. When magenta and green display as white when superimposed. Arrowheads, vasa recta α‐SMA+ perivascular cells. Figure S3. Co‐localizations in Figure 1B. A, 5‐color image from Figure 1C. B, Same image. All possible pairwise combinations of channels are shown. Column titles indicate the channel that is in magenta in that column, and row titles indicate the channel that is in green in that row. When superimposed, magenta and green display as white. Arrowhead, arteriolar pericytes. Dashed line, boundary of glomerulus. Figure S4. CD146 and α‐SMA label perivascular cells in human kidney. Human kidney biopsy tissue was labeled for CD146 (green), α‐SMA (red), nuclei (DAPI, grays) and endothelial cells (Ulex europaeus agglutinin, blue). A, small caliber vessel. Pericytes are double positive for CD146 and α‐SMA (arrowheads). Endothelial cells have detectable CD146 labeling (arrow). Scale bar = 10 μm. B, As well as CD146+α‐SMA+ pericytes (notched arrowhead), pericytes single positive for CD146 (arrow) are also observed. α‐SMA single positive cells (arrowhead) are always located basally to the CD146+ layer. Bar = 15 μm. C, Non‐fibrotic region. Double positive pericytes (arrows) and occasional CD146−α‐SMA+ interstitial fibroblasts are observed. Bar = 25 μm. D, Area of fibrosis. C [file SCT3-10-1232-s002.pdf]

Table S1 Properties of perivascular cell surface markers and their previous use in murine renal studies.

Interstitial Surface Marker	Other names	Description	Use in murine renal studies
PDGFR-α	CD140a	Receptor for PDGF-A, -B & -C.[72] Linked to a fibrotic interstitial cell phenotype in studies of muscle.[35,36]	Studies of glomerular and interstitial fibrosis [78]
PDGFR-β	CD140b	Receptor for PDGF-A, -B & -D.[72] Commonly used pericyte marker in multiple tissues including kidney. Involved in pericyte recruitment during angiogenesis/vasculogenesis. Brain studies suggest that PDGFR-B dependent binding to CD146 in pericyte progenitors facilitates their coverage of endothelial cells.[43]	Common pericyte marker. Studies of glomerular and interstitial fibrosis [78]
CD146	Melanoma-associated Cell Adhesion Molecule (MCAM)	Receptor for Laminin-α-4 (endothelial basement membrane),[50] and interacts with actin cytoskeleton [79] and calcium signalling.[80] Can dimerise with VEGFR2 [81] and PDGFR-β (see above) for angiogenesis roles. Historically used as an endothelial maker; ubiquitous marker of pericytes in human tissue.[27]	Rarely used [25]
NG2	Chondroitin Sulphate Proteoglycan 4	Proposed roles in detecting extracellular matrix components and relaying signals to the cytoskeleton.[82] Necessary for full pericyte coverage of retinal vessels.[83] In human it is specifically not expressed on venular pericytes.[27] Expression lost during pericyte quiescence and regained upon stimulation (e.g. following injury).[24]	Common pericyte marker
α-SMA	Alpha-actin-2	Role in cell contraction. Presence on stromal cells indicates a collagen producing myofibroblast.[84] Also present on contractile pericytes, e.g. on arterioles and descending *vasa recta*.[17]	Extensively used as myofibroblast marker

For Peer Review

Table S2 Proportion of cells in each cluster of the tabula muris senis dataset, and all clusters combined, that is derived from each age group.

**Age group**	**All clusters combined**	**Pericyte Cluster**	**Myofibroblast Cluster**	**Fibroblast Cluster**
All Young (1-3 months)	*48.8%*	*71.9%*	*17.7%*	*60.9%*
1 month	28.0%	43.8%	9.2%	32.2%
3 months	20.8%	28.1%	8.5%	28.7%
All Old (18-30 months)	*51.3%*	*28.1%*	*82.3%*	*39.1%*
18 months	14.8%	10.6%	22.2%	9.2%
21 months	9.3%	11.9%	7.2%	8.0%
30 months	27.3%	5.6%	52.9%	21.8%

For Peer Review

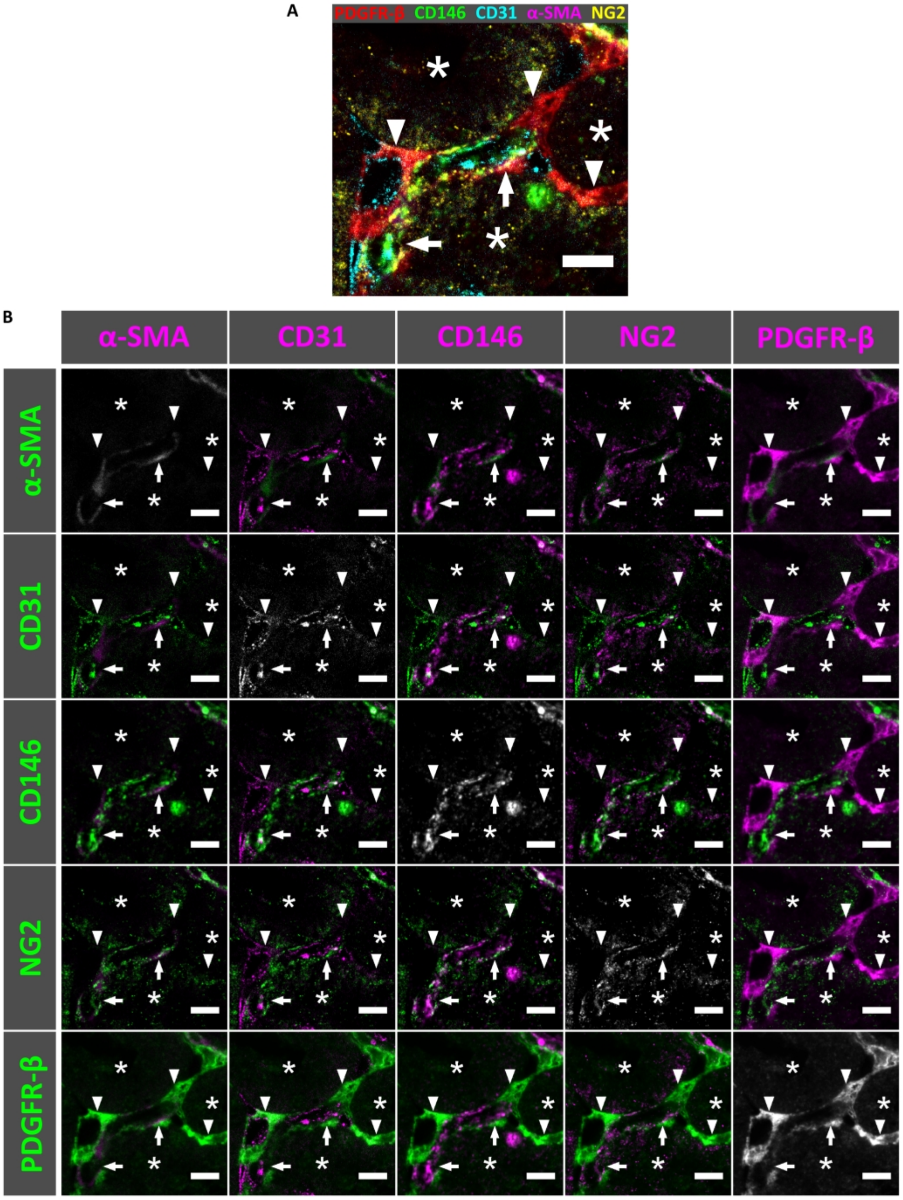

Figure S1 Co-localisations in Figure 1, Panel AA) 5-colour image from Figure 1, Panel A. B) Same image. All possible pairwise combinations of channels are shown. Column titles indicate the channel that is in magenta in that column, and row titles indicate the channel that is in green in that row. Magenta and green display as white when superimposed. Arrows, CD31^{+} capillaries. Arrowheads, PDGFR-β^{+} interstitial cells. Asterisk, a tubule. Scale bar = 10μm.

1924x2561mm (72 x 72 DPI)

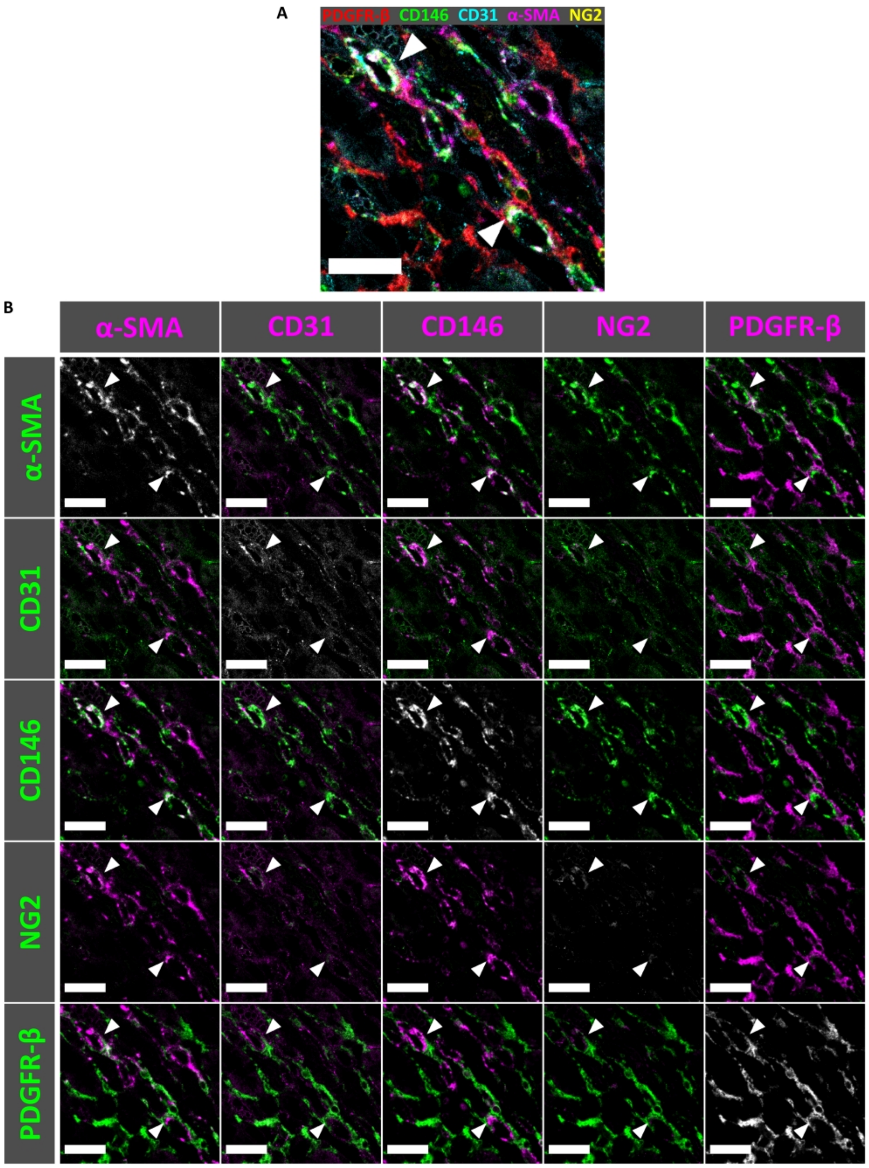

Figure S2 Co-localisations in Figure 1, Panel CA) 5-colour image from Figure 1, Panel C. B) Same image. All possible pairwise combinations of channels are shown. Column titles indicate the channel that is in magenta in that column, and row titles indicate the channel that is in green in that row. Magenta and green display as white when superimposed. Arrowheads, *vasa recta* α-SMA$^+$ perivascular cells. Scale bar = 10μm.

1860x2584mm (72 x 72 DPI)

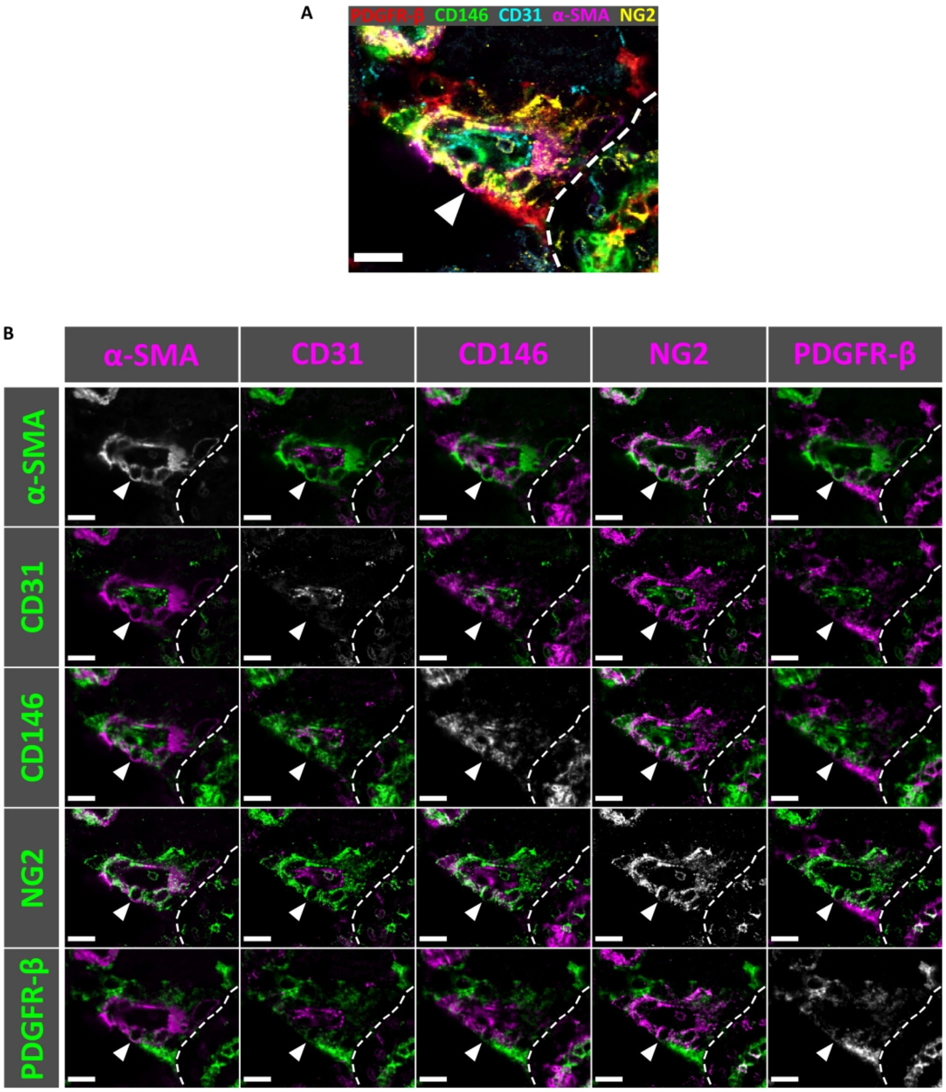

Figure S3 Co-localisations in Figure 1, Panel BA) 5-colour image from Figure 1, Panel C. B) Same image. All possible pairwise combinations of channels are shown. Column titles indicate the channel that is in magenta in that column, and row titles indicate the channel that is in green in that row. When superimposed, magenta and green display as white. Arrowhead, arteriolar pericytes. Dashed line, boundary of glomerulus. Scale bar = 10μm.

2041x2331mm (72 x 72 DPI)

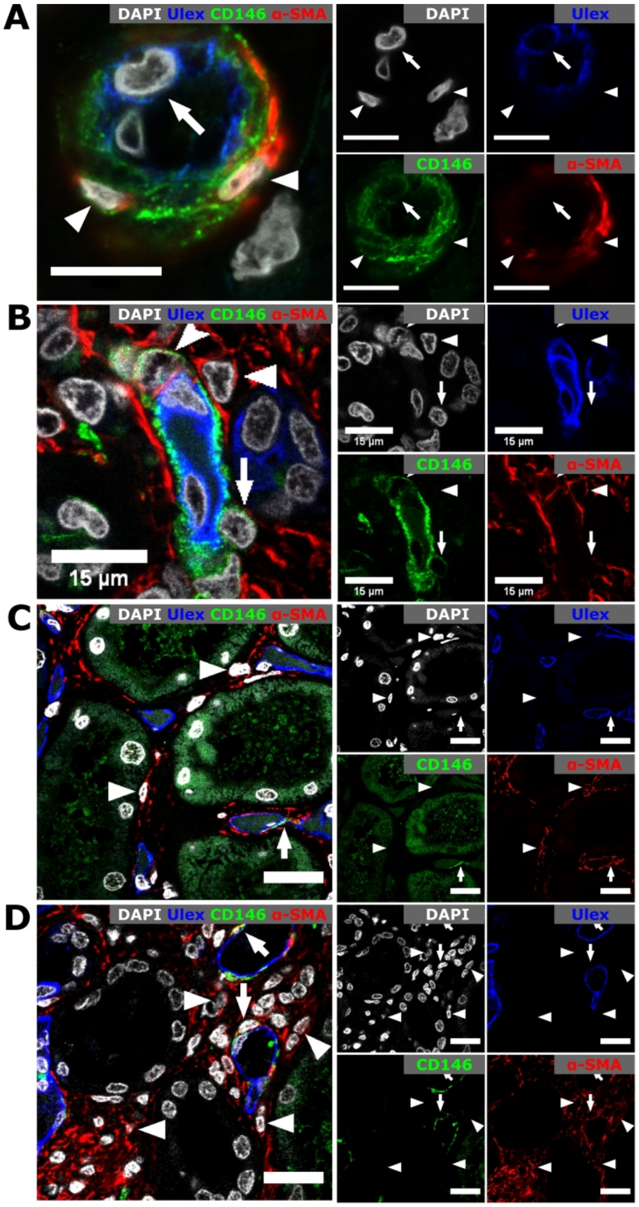

Figure S4: CD146 and ɑ-SMA label perivascular cells in human kidney.%"%"Human kidney biopsy tissue was labelled for CD146 (green), ɑ-SMA (red), nuclei (DAPI, greys) and endothelial cells (*Ulex europaeus* agglutinin, blue). (A) small calibre vessel. Pericytes are double positive for CD146 and ɑ SMA (arrowheads). Endothelial cells have detectable CD146 labelling (arrow). Scale bar = 10µm. (B) As well as CD146$^+$ɑ-SMA$^+$ pericytes (notched arrowhead), pericytes single positive for CD146 (arrow) are also observed. ɑ-SMA single positive cells (arrowhead) are always located basally to the CD146$^+$ layer. Bar = 15µm. (C) Non-fibrotic region. Double positive pericytes (arrows) and occasional CD146-ɑ$^-$SMA$^+$ interstitial fibroblasts are observed. Bar = 25µm. (D) Area of fibrosis. CD146$^+$ɑ-SMA$^+$ pericytes are closely associated with vessels (arrows). There has been an expansion of interstitial fibroblasts, but these are all CD146-ɑ$^-$SMA$^+$. Bar = 25µm.

817x1467mm (72 x 72 DPI)

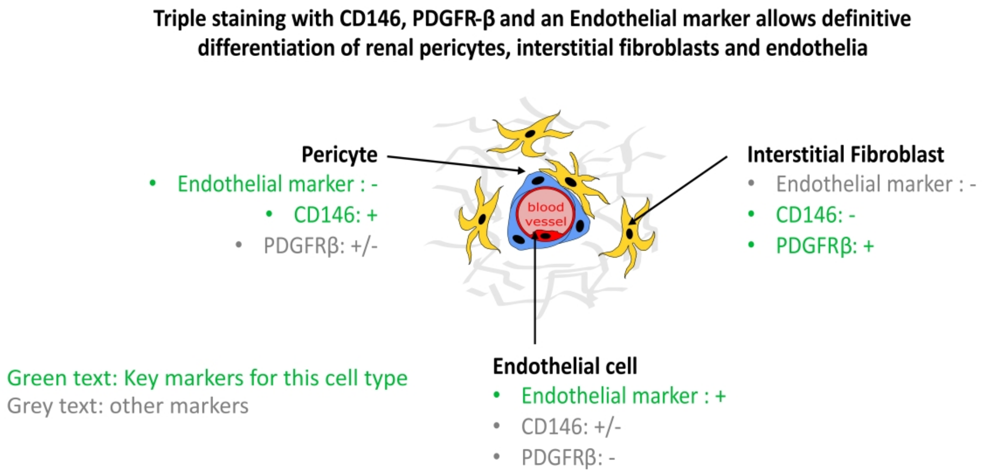

Figure S5 Schematic detailing how to use CD146, PDGFR-β and an endothelial marker to identify pericytes and interstitial fibroblasts in murine kidney.

2012x1111mm (72 x 72 DPI)

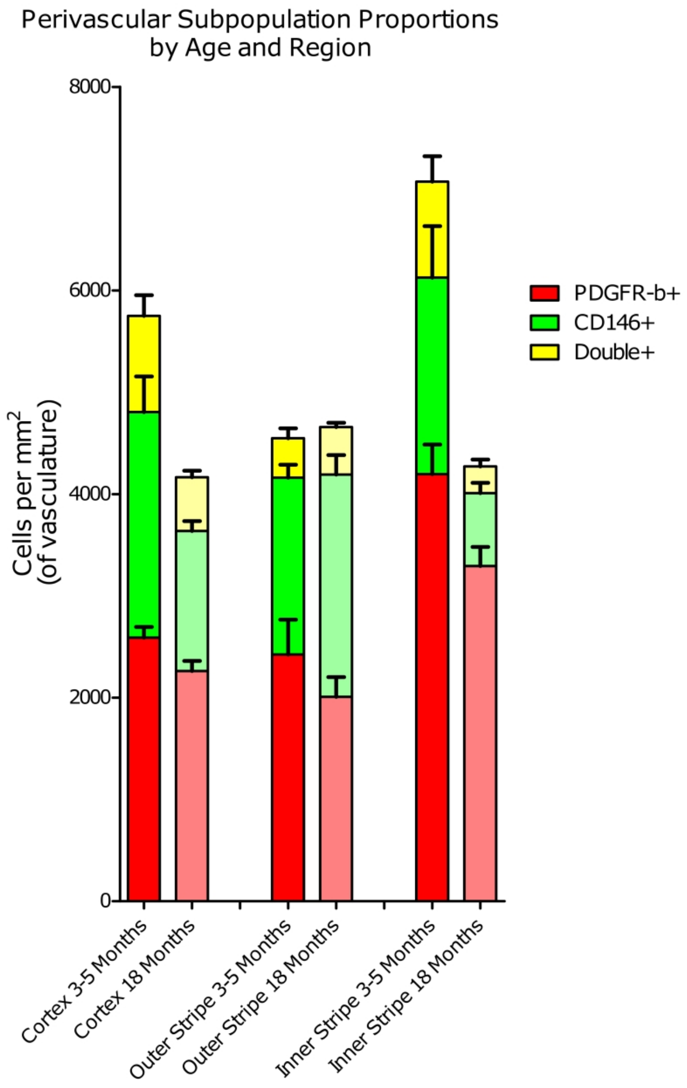

Figure S6 Stacked display of interstitial cell quantification data from Figure 2. Fainter colours indicate data from aged animals. This display highlights how the proportion of PDGFR-β$^+$ cells that are CD146$^-$ (red) far outweighs the proportion that are CD146$^+$ (yellow). If CD146 positivity (in a perivascular location) identifies a pericyte, as proposed, then the majority of PDGFR-β$^+$ cells in the renal interstitium are not pericytes.

1474x1811mm (72 x 72 DPI)

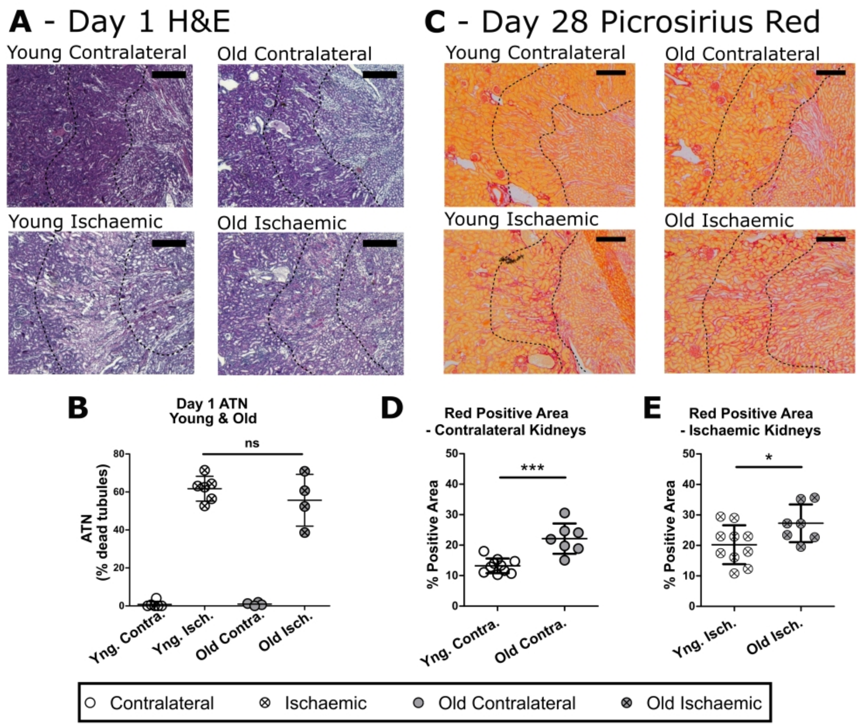

Figure S7: Histological injury scoring at day one and fibrosis scoring at day 28 following unilateral ischaemia reperfusion injury.%"%"A) Low magnification views of day 1 contralateral and ischaemic kidneys of both ages, as indicated, stained with haematoxylin and eosin (H&E). Dashed line outlines the outer stripe. Bar = 500µm. B) Quantification of acute tubular nephropathy (ATN), defined as the percentage of tubules in the outer stripe exhibiting necrotic epithelial cells, at day one post injury. Ischaemic groups compared by Student's t test and no difference detected. C) Low magnification views of picrosirius red (PSR) stained kidneys at day 28 following injury. Dashed line outlines the outer stripe. Bar = 500µm. C,D) Quantification of % red positive area following PSR staining in young and old contralateral (D), and ischaemic (E), kidneys. The old kidneys are more scarred in both cases. Compared using unpaired two-way t test, * $p < 0.05$; *** $p < 0.001$. N = 4-10 per group.

2501x1989mm (72 x 72 DPI)

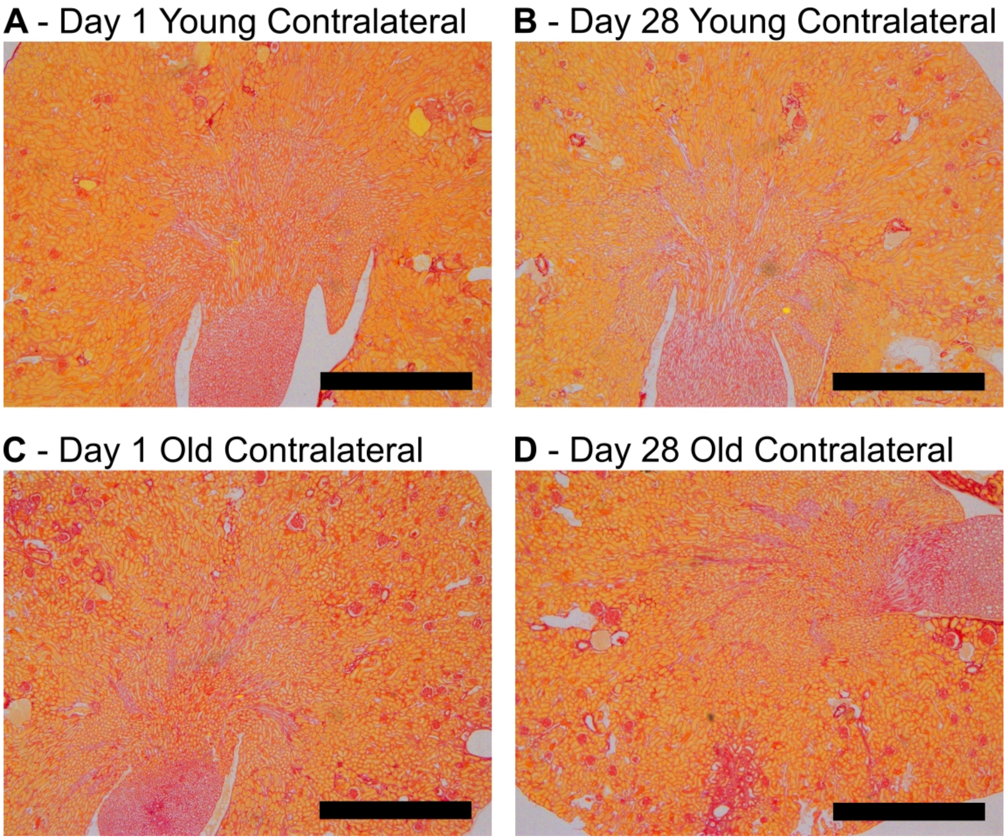

Figure S8: Comparison of fibrosis in contralateral kidneys at days one and 28.%"%"Low magnification representative views of PSR stained sections of contralateral kidney from days one (A,C) and 28 (B,D) post-injury. There is no obvious difference in fibrosis between days in either young (A,B) or old (C,D) groups. Scale bars = 1mm.

2364x1961mm (72 x 72 DPI)

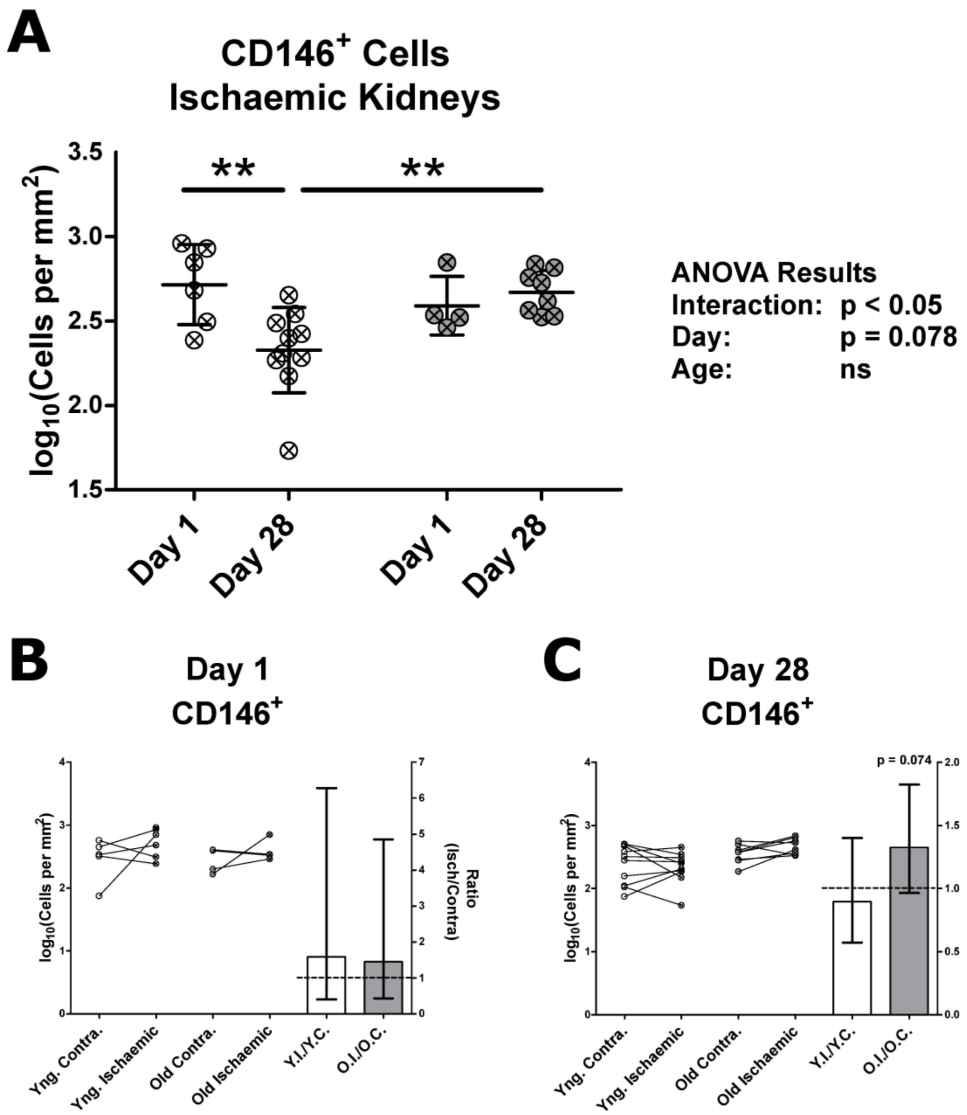

Figure S9: Quantification of CD146$^+$PDGFR-β$^-$ cells in the outer stripe at one and 28 days post-ischaemia.%"%"A) CD146$^+$PDGFR-β$^-$ cell numbers in young and old ischaemic kidneys at day one and 28 post-ischaemia. Groups compared by two-way ANOVA, results displayed. ** $p < 0.01$ in Bonferroni post hoc tests. (B,C) The ratio of CD146$^+$PDGFR-β$^-$ cells between contralateral and ischaemic kidneys is shown at day one (B), and day 28 (C). Bars show mean ± 95% CI. Log ratios were tested for their difference from 0 using a one sample t test. Although CD146$^+$ cell numbers appear decreased in young at day 28 vs day 1 (A), there is no difference compared to the contralateral kidney at either time point (B,C). Likewise, old day-28 ischaemic kidneys have significantly more CD146$^+$ cells compared to the respective young group, but there is no significant difference to the contralateral old kidneys at this timepoint. The differences observed in the ANOVA are thus likely due to the chance interaction of multiple smaller undetectable differences. N = 4-10 per group.

140x155mm (600 x 600 DPI)

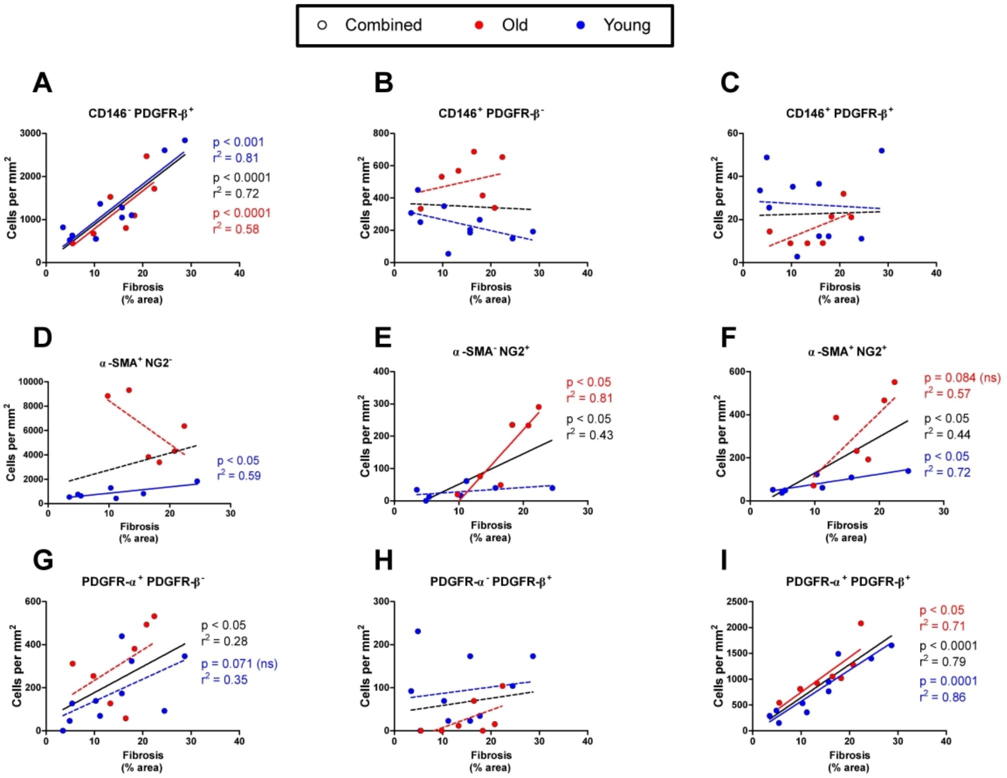

Figure S10: Correlation of perivascular subtype numbers with area of fibrosis in young and old day 28 post-injury kidneys.%"%"Cell frequency is plotted against % fibrosis area. Blue dots and lines correspond to young animals, red dots and lines correspond to old animals, and black lines are for the combined populations. Solid lines indicate a significant relationship. R^2- and p-values are given for significant relationships ($p < 0.05$), and also for non-significant relationships where $p < 0.1$. A-C) Values from CD146 and PDGFR-β dual labelling. D-F) Values from α-SMA and NG2 dual labelling. G-I) Values from PDGFR-α and -β dual labelling. Relationships analysed by linear regression. This plot contains data from Figures 3, 4, and 5.

249x192mm (600 x 600 DPI)

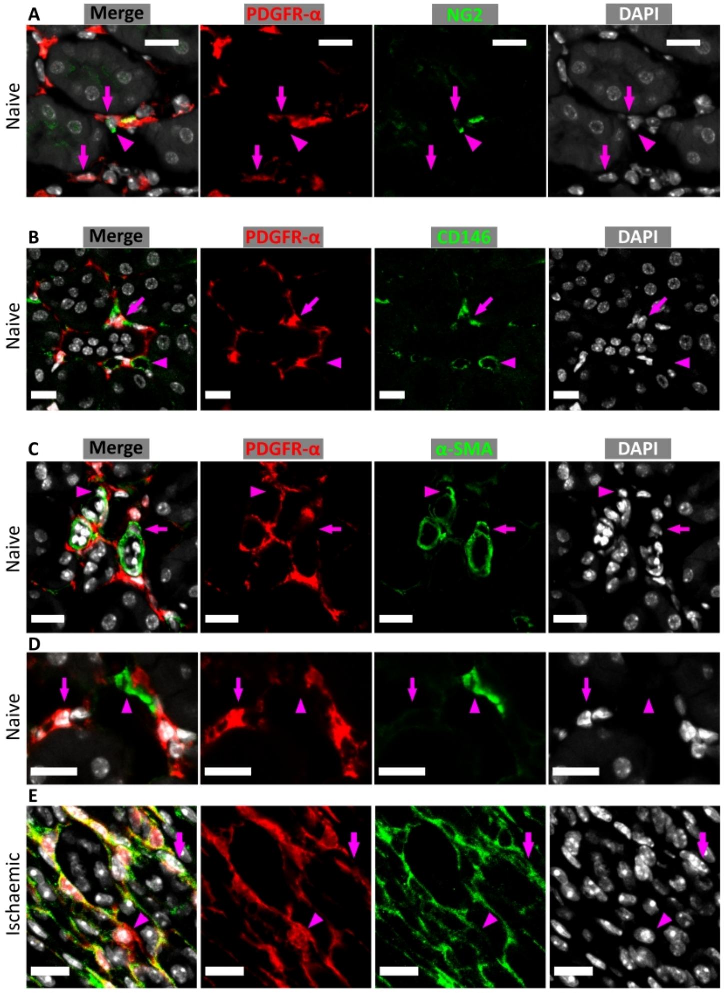

Figure S11 Co-labelling of PDGFR-α with NG2, CD146 and α-SMA in healthy and injured kidney. Kidney from either healthy (A-D) or 28 days post-IRI kidney (E) was labelled for PDGFR-α and either NG2 (A), CD146 (B), or α-SMA (C-E). A) Little interstitial co-localisation of NG2 with PDGFR-α is observed. Single positives for NG2 (arrowheads) and PDGFR-α (arrows) are indicated. B) Little to no co-localisation of CD146 with PDGFR-α is observed. Interstitial PDGFR-α labelling (arrows) and perivascular CD146 labelling (arrowheads) is indicated. C, D) Little co-localisation of PDGFR-α (D, arrow) with α-SMA in healthy tissue, either in α-SMA$^+$ arterioles (C, arrow) or interstitial cells (C,D, arrowheads). E) In 28 days post-IRI tissue, there is extensive co-localisation of α-SMA and PDGFR-α. However, single positive cells for PDGFR-α (arrowhead) and α-SMA (arrow) are also observed. Scale bars 15μm.

1438x2381mm (72 x 72 DPI)

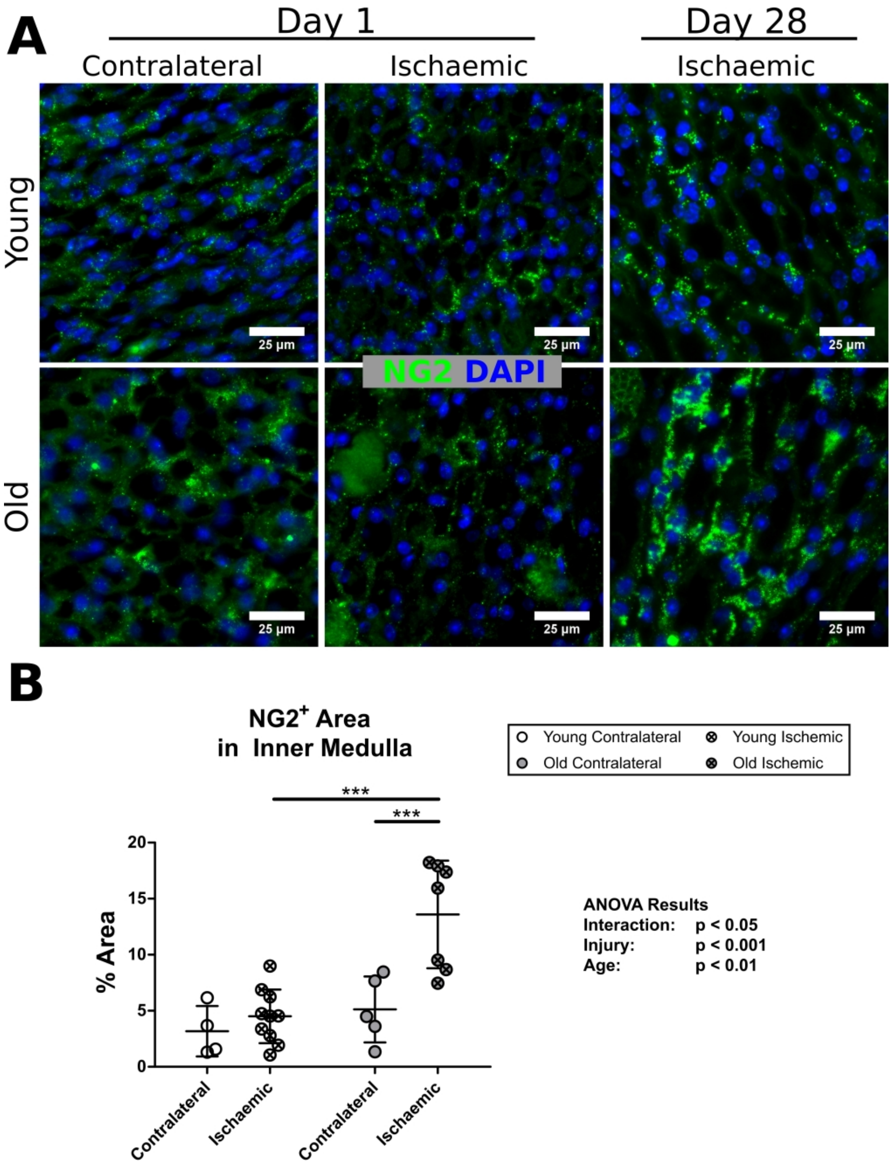

Figure S12: NG2 labelling in the inner medulla.%"%"A) NG2 expression in the inner medulla of young and old contralateral kidney, and day 28 ischaemic kidneys. Scale bar = 25µm. B) Quantification of $NG2^+$ area in the interstitium at day 28 post-IRI. Analysed by two-way ANOVA, *** $p < 0.001$ in Bonferroni post hoc tests. Bar = 25µm. N = 4-10 per group.

1910x2589mm (72 x 72 DPI)

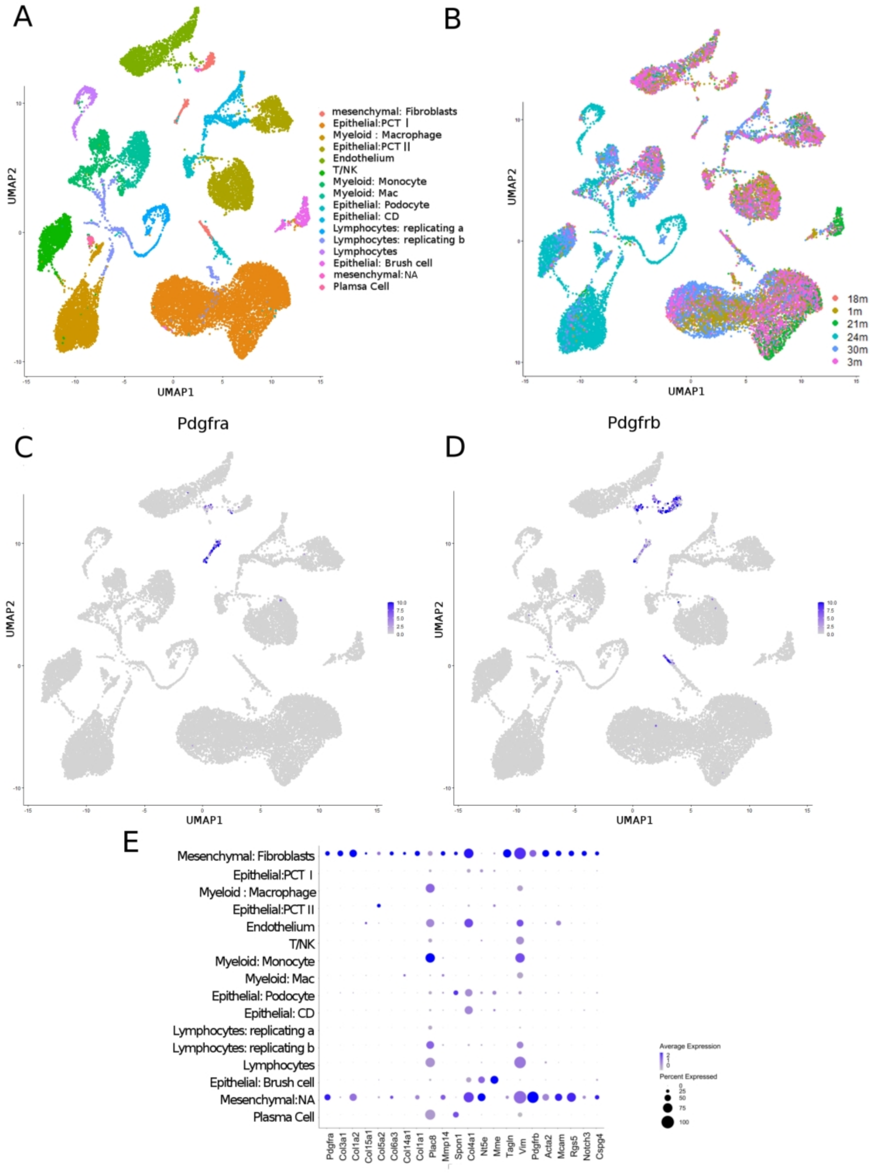

Figure S13: Context of mesenchymal populations within the Tabula Muris Senis single cell RNA sequencing dataset.%"%"A-D) Umap of 21,647 individual transcriptomes from the droplet based kidney analysis within the Tabula Muris Senis dataset[36] coloured by (A) shared nearest neighbour (SNN) allocated cluster; (B) age group; (C) $\log_{10}$ *Pdgfra* expression level; (D) $\log_{10}$ *Pdgfrb* expression level. E) Expression of selected typical mesenchymal genes across clusters. Dot size represents the percentage of cells in each cluster expressing the gene, colour represents average gene expression $\log_{10}$.

1925x2585mm (72 x 72 DPI)

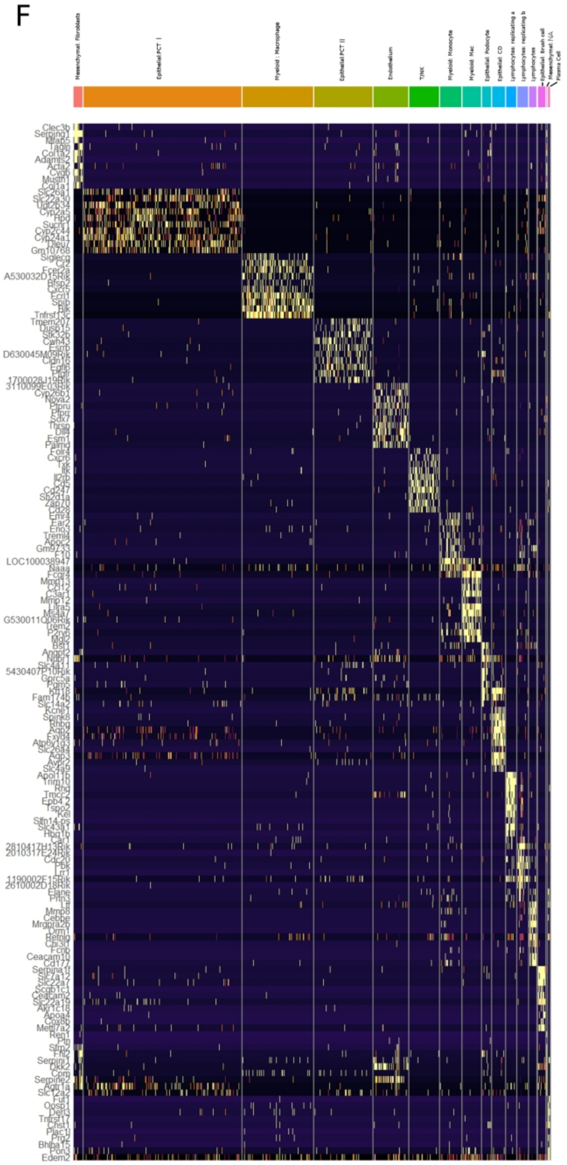

Figure S14: Differentially expressed genes in Tabula Muris Senis dataset clusters.%"%"Heatmap of top 10 marker genes by fold change in each cluster calculated using Wilcoxon signed-rank test. The colour scheme is based on z-score distribution.

1600x3108mm (72 x 72 DPI)

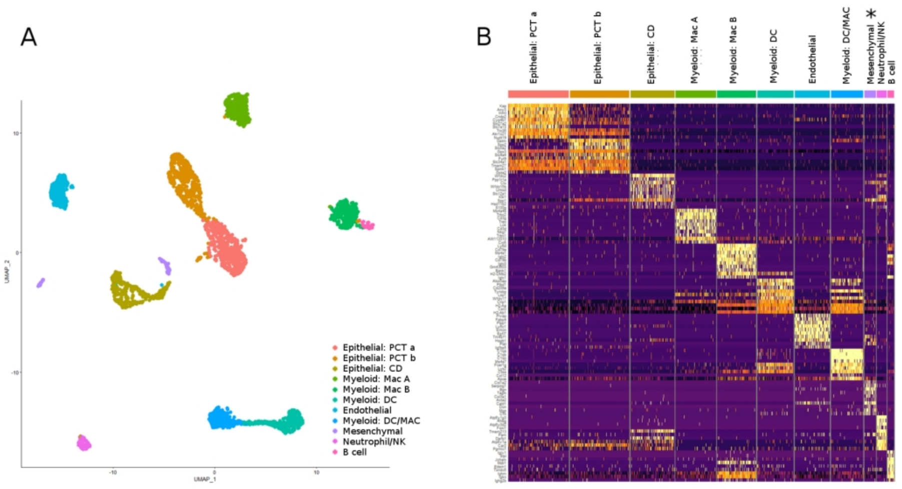

Figure S15: Characteristics of single cell RNA sequencing of whole kidney 28 days post-IRI.%"%"A) Umap of 2931 single cell transcriptomes coloured by shared nearest neighbour (SNN) allocated cluster. B) Heatmap demonstrating the top 10 differentially expressed genes by fold change per cluster, calculated using Wilcoxon signed-rank test. The colour scheme is based on z-score distribution. Asterisk denotes cluster 8, which was taken forward for further sub-clustering to identify mesenchymal cells.

2361x1537mm (72 x 72 DPI)

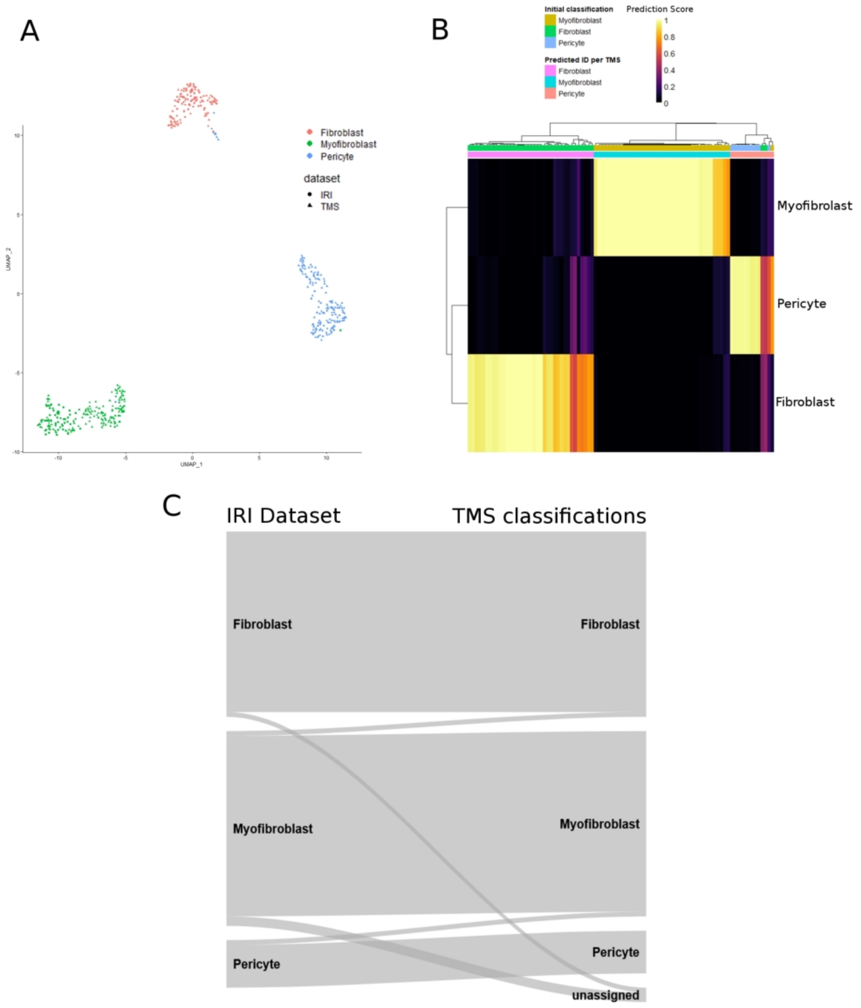

Figure S16: Analysis of cluster equivalence between Tabula Muris Senis dataset and post-IRI dataset. %"%"A) UMAP of 490 single cells from integrated IRI and TMS dataset. Coloured by initially assigned classification in the individual datasets, shape shows dataset of origin. B) Heatmap showing classification prediction score for each cell in the IRI dataset based on transcriptional anchors derived from TMS classifications. C) Sankey diagram demonstrating the mapping of cells within each initial cluster of the IRI dataset to predicted classifications based on the TMS dataset, as calculated by SCMAP.

1925x2584mm (72 x 72 DPI)

Supplemental Methods

Animals and Surgery

For ischaemia reperfusion injury (IRI) surgery, anaesthesia was induced and maintained using 2-4% isofluorane (Merial). Temperature was maintained at 37°C using a homeostatically controlled blanket (Harvard Apparatus, Boston MA). A flank incision was made and the left renal pedical exposed and occluded by atraumatic clamp. Ischaemia was thus maintained for 25 minutes (FVB mice) or 20 minute (C57Bl/6 mice) before clamp removal. Ischaemia time is titrated to each mouse strain to cause an equivalent ischaemic injury. The peritoneum was sutured and the skin stapled. 1ml sterile saline was administered subcutaneously post-surgery along with Buprenorphine analgesia and the animals were maintained in an incubator overnight. Animals were culled one or 28 days post-ischaemia by exsanguination and cervical dislocation, then perfused with cold PBS through the left ventricle until the fluid ran clear.

Immunofluorescence Staining

The following antigens were visualised using secondary antibody staining: α-SMA (1:500, Sigma, A2547); CD31 (1:25, Dianova, DIA-310); CD34 (1:100, Abcam, ab81289); NG2 (1:50, Millipore, Ab5320); PDGFR-α (1:75, Cell Signalling, 3174S). For human endothelial staining biotinylated *Ulex europaeus* Agglutinin I (Vector, B-1065) was used. Briefly, 5μm slides were dewaxed and rehydrated, then boiled for eight minutes in a 10mM citrate buffer pH6.0 in a microwave. After cooling slides were blocked for 10 minutes in Protein Block (DAKO) washed in PBS then blocked for 1h at room temperature (RT) in 5% serum (matching the species of the secondary antibody). Primary antibodies were diluted in 5% serum and incubated with slides overnight at 4°C. After washing in PBS the subsequent day, slides were incubated with species-matched fluorochrome conjugated secondary antibody (1:250, Invitrogen) for 2h at RT. For human endothelial staining, fluorochrome-conjugated streptavidin (Invitrogen) was used. After washing slides were counterstained with DAPI for 10 minutes before mounting with coverslips. CD146 (1:750, Abcam, ab75769) and PDGFR-β (1:500,

For Peer Review

Abcam, ab32570), both raised in rabbit, were visualised using TSA-Plus tyramide reagents (Fluorescein, Cyanine 3, or Cyanine 5; PerkinElmer) by the above method with the following amendments: Blocking steps included a 10min incubation in 3% H_2O_2 (Sigma) in PBS. Following incubation of the first primary antibody, 2 drops of ImmPRESS anti-rabbit secondary antibody (Vector) were applied for 30min at RT. After washing, slides were incubated in the tyramide reagent for 7 minutes to visualise, then washed in deionised water. Slides were then boiled for 6 minutes in 10mM citrate buffer pH6.0 (to denature antibodies), blocked with 3% H_2O_2 for 15 minutes (to deactivate peroxidase enzyme), then labelling with the second primary antibody proceeded as normal. For 5-colour immunostaining this procedure was repeated twice for CD146 and PDGFR-β, CD31 was visualised using BV480-conjugated Goat anti-Rat (1:250, BD Biosciences, 564878), α-SMA was visualised using BV421-conjugated Goat anti-Mouse (1:250, BD Biosciences, 563846) and NG2 was visualised last with Alexafluor 555-conjugated Goat anti-Rabbit (1:200, Invitrogen, A21428).

Single cell transcriptomics analysis and statistics

A similar workflow was used for the IRI and TMS dataset, following a standard sequence of procedures of filtering, highly variable gene selection, dimensionality reduction and clustering were performed using the scRNAseq analysis R package Seurat(v3)2. Following alignment and pre-processing, we filtered out any features not present in at least 3 cells and any cells without at least 200 features and were left with 15340 features (genes) across 3562 cells in the IRI dataset and 20138 features across 21647 cells I the TMS dataset.

To exclude low-quality cells and begin to exclude probable doublets, we filtered any cells that expressed fewer than 200 genes or over 2000 and with at least 500 unique molecular identifiers but fewer than 10000. We used a mitochondrial filter to remove cells in which >= 50% of genes were mitochondrial. This is a higher filter than has been used in non-renal cell single cell analysis but given the high metabolic demands of renal tubular epithelial cells, we found that our cells expressed a lot of mitochondrial RNA and it was of limited utility as a QC metric. A similar cut-off has been used in other renal specific single cell sequencing. Any gene not expressed in at least 3 cells was removed. At

For Peer Review

this point we were left with 15340 genes across 2931 cells in the IRI dataset. The TMS data was pre-processed according to the original manuscript prior to access so this step was skipped.

Normalisation was performed using the Seurat package to reduce biases introduced by technical variation, sequencing depth and capture efficiency. We employed the default global-scaling normalization method “logNormalize” which normalised gene expression per cell by the total expression and multiplies the result by a scaling factor before log transformation.

The expression matrix subsequently underwent dimensionality reduction using principal component (PC) analysis of the highly variable genes within the dataset. Using Seurat’s FindVariableGenes function (and computed using the “vst”) which models the mean-variance relationship we calculated the top 2000 highly variable genes. We then scaled the data to shift the variance across cells to 1. PC analysis was performed using these selected genes and 20 PCs were identified for subsequent analysis in each dataset, selected both visually using the elbow point on the bowplot and via the Jackstraw method.

Clusters were then assigned using a K-means shared nearest neighbour-based algorithm using Seurat’s FindClusters function, built using the first 20 PCs and a resolution parameter of 0.5. The original Louvain modularity optimization algorithm was employed. t-Distributed stochastic neighbour embedding (tSNE) (using the Rtsne package Barnes-Hut implementation) was performed with the resolution set to 0.5. Dimension reduction and visualisation of the data was then performed using the UMAP algorithm using the first 10 PCs as an input and default settings.

For all single-cell differential expression tests, we used the Wilcoxon rank sum test to identify a unique expression profile for each cluster, differential expression was tested between each cluster and all other clusters combined. The FindAllMarkers test as implemented in Seurat using a “min.pct” of “0.25” and a “logfc.threshold” of 0.25 returns an “adj_pval” (Bonferroni adjusted p-values) and an “avg_logFC” (average log fold change) for each gene. Genes were ranked in order of average log fold change and visualised using heatmaps. This allowed classification based on the literature and our own knowledge of renal single cell gene expression.

For Peer Review

Any candidate stromal/mesenchymal cell cluster was identified within the data, subsetted out, and renormalized, scaled, and clustered as before. This allowed some stray non-mesenchymal cells to be pruned from the data, leaving a high confidence mesenchymal subset of 90 cells for further analysis. The Final UMAP plot as seen in Figure 8 was calculated using the first 20 PCs with Seruats RunUMAP function, with the arguments pertaining to number of neighbours set to 8, a minimum distance of 0.001 and a spread of 4. The clusters were calculated on the first 10 PCs with a resolution of 1.

To test whether our classification of cells in the TMS datasets would apply accurately to the cells in the IRI dataset we then integrated using the “anchoring” approach introduced in Seurat v3 . Here we create an integrated reference dataset and transfer the cell type labels from the TMS dataset onto the IRI cells. Briefly, this approach requires identification of “anchors” between the datasets which represent shared biological states. This involves jointly reducing the dimensionality of both datasets using diagonalized canonical correlational analysis before searching for mutual nearest neighbours in the new shared space. The paired cells are treated as anchors which represented shared biology across the datasets. Anchors were identified using the default parameters of the “FindIntegrationAnchors” function, with the argument dims=1:20. Following this 89/90 cells in the IRI data were assigned a classification which corresponded exactly to the classification we had manually assigned “de-novo”. (Figure S10A,B). Additionally, we used the SCMAP approached to project cells classifications from the TMS scRNA-seq data set onto individual cells from the IRI data. The mapping is shown in the Sankey plot, showing good concordance (Figure S10C).

For Peer Review

Animal Experimental Series

Animal Series	Mouse Strain	Intervention	Sample Size	Outcome measures	Blinding	Figure refs
**Ageing naïve**	FVB	Natural ageing	Young: 5<br>Old: 9	Immunostaining	Blind during analysis	1, 2, S2
**Ageing uIRI**	FVB	Natural ageing, uIRI	Young day 1: 6<br>Old day 1: 4<br>Young day 28: 10<br>Old day 28: 8	Pathology, Immunostaining	Blind during analysis	3 – 5, S3 – S7
**scRNAseq**	C57Bl/6JN	None/Ageing	Age months : n number (1:2, 3:11, 18:6, 21:3, 30:4)	Single cell transcriptomics	Blind to age during analysis	6, S8, S9, S11
**scRNAseq**	C57Bl/6	uIRI	3 per group	Single cell transcriptomics	none	7, S10, S11

For Peer Review
